# An Anti-β-Amyloid Vaccine for Treating Cognitive Deficits in a Mouse Model of Down Syndrome

**DOI:** 10.1371/journal.pone.0152471

**Published:** 2016-03-29

**Authors:** Pavel V. Belichenko, Rime Madani, Lorianne Rey-Bellet, Maria Pihlgren, Ann Becker, Adeline Plassard, Stephanie Vuillermot, Valérie Giriens, Rachel L. Nosheny, Alexander M. Kleschevnikov, Janice S. Valletta, Sara K. S. Bengtsson, Gordon R. Linke, Michael T. Maloney, David T. Hickman, Pedro Reis, Anne Granet, Dorin Mlaki, Maria Pilar Lopez-Deber, Long Do, Nishant Singhal, Eliezer Masliah, Matthew L. Pearn, Andrea Pfeifer, Andreas Muhs, William C. Mobley

**Affiliations:** 1 Department of Neurosciences, School of Medicine, University of California San Diego, La Jolla, California, United States of America; 2 AC Immune SA, Lausanne, Switzerland; 3 Department of Neurology and Neurological Sciences, Stanford Medical School, Stanford, California, United States of America; IGBMC/ICS, FRANCE

## Abstract

In Down syndrome (DS) or trisomy of chromosome 21, the β-amyloid (Aβ) peptide product of the amyloid precursor protein (APP) is present in excess. Evidence points to increased *APP* gene dose and Aβ as playing a critical role in cognitive difficulties experienced by people with DS. Particularly, Aβ is linked to the late-life emergence of dementia as associated with neuropathological markers of Alzheimer’s disease (AD). At present, no treatment targets Aβ–related pathogenesis in people with DS. Herein we used a vaccine containing the Aβ 1–15 peptide embedded into liposomes together with the adjuvant monophosphoryl lipid A (MPLA). Ts65Dn mice, a model of DS, were immunized with the anti-Aβ vaccine at 5 months of age and were examined for cognitive measures at 8 months of age. The status of basal forebrain cholinergic neurons and brain levels of APP and its proteolytic products were measured. Immunization of Ts65Dn mice resulted in robust anti-Aβ IgG titers, demonstrating the ability of the vaccine to break self-tolerance. The vaccine-induced antibodies reacted with Aβ without detectable binding to either APP or its C-terminal fragments. Vaccination of Ts65Dn mice resulted in a modest, but non-significant reduction in brain Aβ levels relative to vehicle-treated Ts65Dn mice, resulting in similar levels of Aβ as diploid (2N) mice. Importantly, vaccinated Ts65Dn mice showed resolution of memory deficits in the novel object recognition and contextual fear conditioning tests, as well as reduction of cholinergic neuron atrophy. No treatment adverse effects were observed; vaccine did not result in inflammation, cellular infiltration, or hemorrhage. These data are the first to show that an anti-Aβ immunotherapeutic approach may act to target Aβ-related pathology in a mouse model of DS.

## Introduction

Down syndrome (DS), or trisomy 21, affects one in 733 newborns [1,2,3,4]. In addition to cognitive dysfunction during childhood, those with DS are predisposed to Alzheimer disease (AD). Mature neuritic plaques and neurofibrillary tangles are present by age 40 in DS [5] and about 60% have dementia by age ~60 [6,7]—i.e. approximately 25 years earlier than those with late onset AD. Among human chromosome 21 (HSA 21) genes, increased dose of the gene for APP and the β-amyloid (Aβ) have been shown to be necessary for the emergence of AD-like symptoms in DS [8,9]. Outside the DS context, APP gene dose is sufficient to cause AD, as demonstrated in several families harboring a duplication of the APP gene [10,11]. The age of onset of dementia in these families is similar to DS [12,13]. Thus, increased APP gene dose is necessary for AD-like neuropathology in DS and sufficient in those without DS.

Longevity in DS has increased from an average of 9 years in 1933 [9] to approximately 60 years [14,15]. With longer life has come increased risk for AD-like symptoms. Drugs proven effective for treating cognitive symptoms in AD, including cholinesterase inhibitors and Memantine, have demonstrated no significant clinical benefit in DS [16,17,18] (reviewed in [19]). As for AD, it has been suggested that targeting APP processing or Aβ levels may hold promise. Immunotherapies for sporadic AD have reached the clinic, but as yet conclusive evidence of benefit is lacking [19,20,21]. One factor that may have limited success is the late stage of disease intervention [22,23,24,25]. Ideally, treatments would prevent pathogenesis. For the population at large, this approach awaits development of biomarkers that report on the earliest stages of synaptic dysfunction. In contrast, in early onset familial AD (FAD) and DS the diagnosis and treatment could begin well before advanced pathogenesis. No approach yet addresses this possibility in DS [26].

In support of possible future trials of immunotherapy in DS, we evaluated active immunization against Aβ in a mouse model of DS. Ts65Dn mice, widely used for this purpose, are segmentally trisomic for a portion of mouse chromosome 16 homologous to HSA 21 that contains the murine gene for App [27]. Ts65Dn mice show increased full length murine App and its products, including Aβ40 and Aβ42 [28]. While Ts65Dn mice fail to develop neuritic plaques, congophilic angiopathy or neurofibrillary tangles, changes in synaptic structure and function are present early in life and persist throughout their life [29,30]. Ts65Dn mice demonstrate behavioral deficits in several memory tasks, [31,32] with changes apparent at age 3 months [32]. Deficits in novel object recognition and contextual fear conditioning are evidence of dysfunction of hippocampal circuits. Age-related neuronal dysfunction and degeneration is documented in Ts65Dn mice [33,34]. Importantly, increased App gene dose is necessary for degeneration of locus coeruleus and basal forebrain cholinergic neurons (BFCNs), with atrophy and loss of BFCNs emerging between 6 and 12 months of age. Ts65Dn mice thus serves as a genetic model of DS to examine neurodevelopmental as well as neurodegenerative events.

Herein, the vaccine DS-01, generated using a liposome-technology [35] was used to target mouse Aβ. We investigated whether the vaccine would break Aβ self-tolerance and, if so, improve hippocampal-mediated memory deficits and prevent atrophy of BFCNs without eliciting adverse events, including brain inflammation and hemorrhage.

## Materials and Methods

### Mice

Segmental trisomy 16 (Ts65Dn) male mice [27] were maintained on the B6/C3H background; diploid (2N) littermate mice served as controls. Mice were housed and genotyped as described [32] and prescreened for Pde6brd1 homozygosity, as described [36]. Experiments were conducted in accordance with the National Institutes of Health guidelines under protocols approved by the Stanford University and University of California San Diego (UCSD) Institutional Animal Care and Use Committees.

### DS-01 Vaccine preparation

Tetrapalmitoylated mouse Aβ1-15-peptide (Pal1-15) was prepared by first solubilizing dimyristoyl phosphatidyl choline, dimyristoyl phosphatidyl glycerol, cholesterol and monophosphoryl Lipid A (MPLA) (all from Avanti Polar Lipids, Inc., Alabaster, AL, USA) at molar ratios 9:1:7:0.05 in EtOH (30 minutes at 60°C). The lipid/ethanol solution was diluted in phosphate buffer saline (PBS), pH 7.4, then concentrated via ultrafiltration and dilution by diafiltration in PBS, pH 7.4. The multilamellar liposomes were submitted to homogenization followed by sequential extrusion though polycarbonate filters (Whatman, GE Healthcare, Bottmingen, Switzerland), pore size 0.2 μm using EmulsiFlex-C5 (Avestin, Ottawa, ON, Canada). The liposomes were diluted in PBS, pH 7.4, and heated to 60°C prior to peptide addition. The peptide was dissolved in PBS pH 11.4 with 5% ß-Octylglucoside, injected in the liposome solutions at 60°C and stirred for 30 minutes followed by concentration steps through ultrafiltration and dilution in PBS, pH 7.4, by diafiltration. Finally, the vaccine was filtered by passing through 0.2 μm polycarbonate syringe filters (Sartorius, Stedim Biotech, Goettingen, Germany) and stored at 2–8°C. The peptide to lipid molar ratio was 1:100. Empty liposome vaccine (vehicle) was prepared identically but lacked the peptide.

### Immunizations

Mice, males at age 5 months, received six subcutaneous immunizations (days 1, 14, 28, 42, 56, and 70) with 200 μl of either DS-01 (51.2 μg per dose of Pal1-15) or vehicle. Two cohorts of Ts65Dn (n = 15/each) and control 2N mice (n = 20/each) were used. Tail bleeding was performed before immunization on day 1 and on day 56; plasma samples were separated from blood by centrifugation (4000g 5 minutes at 4°C) without antibody-antigen dissociation. At sacrifice (age 9 months) plasma was taken and brains were dissected [37].

### Quantification of mouse Aβ-specific antibodies

Aβ-specific IgG responses in plasma were determined by ELISA using mouse Aβ42 or Aβ40 (Bachem, Bubendorf, Switzerland) essentially as described^57^ but with 4G8 antibody (1 mg/mL, serially diluted; Covance, Massachusetts, USA) to establish a standard curve. Results were expressed as ng/mL, with reference to 4G8, or as optical density (O.D.) at a plasma dilution of 1:100.

### Defining the immunoreactivity of the vaccine-induced plasma

Brain tissue was homogenized as described [38] and immunoblottings, using 4–12% Bis-Tris Plus Gels or Novex 10–20% Tricine Protein Gels, were performed under reducing conditions according to manufacturer’s instructions (Invitrogen, Illkirch, France). Mouse or human Aβ42 (Bachem, Bubendorf, Switzerland) or recombinant mouse C99 fragment (672–770 residues) (Cloud-Clone Corp, TX, USA) were used as controls. Membranes were blotted with either primary antibodies: rabbit anti-App C-terminal (AHP538, 1:6000, AbD Serotec, Puchheim, Germany) or A8717 (1:6000, Sigma-Aldrich, MO, USA); or with plasma from DS-01-treated Ts65Dn mice (1:100 dilution, blocking buffer/ 0.1% Tween). All membranes were reblotted with mouse α-tubulin (T5168, 1:6,000, Sigma-Aldrich) to normalize protein loading. Secondary antibodies were: goat anti-mouse-IRDye800 and goat anti-rabbit-IRDye680 (1:10,000, Li-Cor Biosciences, NE, USA). Bands were quantified using Li-Cor Odyssey (Li-Cor). Bands of interest were normalized to α-tubulin.

Immunoreactivity was also studied using extracts from cells. Control CHO cells and CHO cells stably expressing human APP with the Indiana mutation (V717F) [39] were cultured in Hams F12, 10% FBS, 1% pen/strep with 500 μg/mL of G418 (Invitrogen, USA). At confluency, cells were harvested in PBS and centrifuged; the pellet was re-suspended in RIPA buffer (1% Triton X-100, 1% NP-40, 0.1% SDS, 0.1% sodium deoxycholate) with 1mM of PMSF, incubated on ice (30 minutes) and centrifuged at 4°C (30 minutes) before collecting the supernatant for immunoblotting [40]. PC12M cells (Dr. M. White, UT Southwestern Medical Center, TX, USA) were cultured as described [41,42] and at a confluency of ~ 70% were transfected with either 4 μg of GFP, *APP*-GFP or C99-GFP plasmid [41] using TurboFect (Thermo Fisher Scientific, USA) following the manufacturer’s protocol. After 24 hours, cell extracts were prepared in RIPA buffer with PMSF and prepared as above for immunoblotting. Immunoblotting was performed according to a standard method [40] using plasma from Ts65Dn mice immunized with DS-01 (diluted 1:1000) or an antibody against the C-terminus of human APP (1:1000) [43]. Detection used HRP secondary antibodies (1:10,000; Santa Cruz Biotech, CA, USA) and chemiluminescence (Amersham Pharmacia Biotech, USA).

### Behavioral testing

At approximately 8 months of age, mice were tested in this order: locomotor activity (starting on day 11 after the last immunization); novel object recognition; and contextual fear conditioning. All behavioral tests were performed during the light cycle between 7:00 a.m. and 7:00 p.m and mice from both genotypes and treatments were tested at the same time of the day. Spontaneous locomotor activity was monitored as previously described [32]. For the novel object recognition test, object preference was examined in prior experiments with mice of similar age, and objects with equal preferences were randomly selected as ‘familiar’ and ‘new’. A protocol with two sample objects was used with a 24 hour delay, as described [32,44]. Contextual and cued fear conditioning was conducted over three days for evaluation of fear-dependent learning and retrieval, as described [32]. This test was performed using chambers from Coulbourn Instruments (Whitehall, USA).

After testing, mice were weighed after deep anesthesia with sodium pentobarbital (200 mg/kg, intraperitoneal injection, Abbott Laboratories, IL, USA). Brains were weighed (with olfactory bulbs and cervical spinal cord through C1–C2) and stored (-80°C) immediately or after transcardial perfusion (4% paraformaldehyde) [45] prior to immersion in 4% paraformaldehyde and storage (4°C).

### Measurements of App mRNA, protein and its products in Ts65Dn mice at postnatal day 21

Total RNA was extracted from cortex of 21-day old mice using RNeasy Kit (Qiagen, Germantown MO, USA) and used for cDNA generation with High-Capacity cDNA Reverse Transcription Kit (Applied Biosystems, Grand Island NY, USA). The primer sequences used for App were as described [33]. For quantification, values were normalized to endogenous glyceraldehyde-3-phosphate dehydrogenase GAPDH using RT^2^ qPCR Primer Assay (Qiagen, Germantown MO, USA). Polymerase chain reaction was for 40 cycles. Values within the log-linear phase of the amplification curve were defined for each probe/primers set and analyzed using the ΔΔCt method (ABI PRISM 7300 Sequence Detection System, user bulletin number 2).

App full length protein, C-terminal fragments (CTFs, including α-CTF C83 and β-CTF C99) and Aβ40 levels were also measured in 21 day old mouse cortex homogenized in RIPA buffer with Protease Inhibitor Cocktail (1x from 100x stock; Sigma Aldrich, St. Louis, MO, USA) prior to measuring protein concentration (Bio-Rad Laboratories, CA,USA). Aβ40 levels were measured using the Aβ Peptide Panel 1 (4G8) V-Plex kit, from MSD (Meso Scale Discovery, Rockville MD, USA) and analyzed according to manufacturer’s instructions. The levels of App full length protein and APP-CTF’s were measured by immunoblotting [40] using 4–12% gels (Invitrogen, CA, USA). The primary antibody affinity-purified rabbit polyclonal antibody to the C-terminus of human APP (1:1000) [43] and the secondary antibody goat anti-rabbit IgG-HRP conjugate (1:10,000) (Jackson Immunoresearch Laboratories Inc; PA, USA) were used. The membranes were imaged using the Bio-Rad ChemiDoc XRS (Bio-Rad Laboratories Inc, CA, USA) following manufacturer’s instructions. Data were analyzed using Image J (integrated density).

### Quantification of the level of Aβ

Brain regions (cortex, hippocampus, cerebellum) were dissected as described in [30] and homogenized in 50 mM NaCl, 0.2% diethylamine at 100 mg wet weight / ml. Extracts were centrifuged (4°C, 1 hour, 100,000×g) and supernatants neutralized with 1/10 volume 0.5 M Tris–HCl pH 6.8 before adding to ELISA plates containing 50 mL EC buffer (0.02 M Na phosphate, pH 7.0 containing 0.002 M NaCl, 0.2% BSA, 0.05% CHAPS, 0.4% Block Ace, 0.05% NaN3). Brain homogenates and plasma were analyzed using sandwich ELISA kits according to the manufacturer’s protocol (Invitrogen, CA, USA).

### Quantification of the level of pro-inflammatory markers and histological examination

Plasma and brain extracts were analyzed for interferon-gamma (IFN), tumor necrosis factor (TNF), interleukin-1 (IL-1) and interleukin-*6* (IL-6) according to the manufacturer’s instructions (MSD) with V-PLEX Plus Mouse Biomarkers kits. Cryostat sections (10 μm) were stained with the Perls' Prussian blue method for detection of iron deposits as described [46]. For detection of lymphocytes, an antibody against mouse CD4 cells (Millipore, Massachusetts, USA) was used for immunostaining [47]. Analysis of neuropathology and the vasculature were carried out using sections stained with hematoxylin and eosin (H&E). Sections were stained in duplicate and blind-coded. Analysis was performed with a digital Olympus BX41 stereomicroscope.

### Immunofluorescent staining of medial septum cholinergic neurons

Brain sections were incubated with either 5% nonfat milk or 5% normal donkey serum in 0.1M PBS overnight (4°C) with one of the following antibodies: rabbit anti-cow glial fibrillary acidic protein (GFAP, (DAKO, Glostrup, Denmark; 1:500), polyclonal rat anti-CD45 (Pharmingen, CA, USA 1:5000), or goat anti-choline acetyltransferase (ChAT) (Millipore; Massachusetts, USA; 1:100). Sections were rinsed in PBS, incubated with species-appropriate biotinylated secondary antibodies (1:200; Jackson ImmunoResearch, PA, USA) (1 hour, 21°C) rinsed in PBS and incubated with fluorescein isothiocyanate-conjugated streptavidin (1:500; Jackson ImmunoResearch, PA, USA) (1 hour), before a final rinse, mounting and coverslipping. To control antibody specificity, no primary antibody was added to selected sections; immunofluorescence was not observed under these conditions.

### Confocal imaging

Slices were examined and scanned in a Radiance 2000 (Bio-Rad, UK) confocal microscope attached to a Nikon Eclipse E800 fluorescence microscope according to manufacturer’s instruction [30]. ImageJ was used to determine: density of ChAT+ cells; area of individual ChAT+ neurons, and average optical density of ChAT in individual neurons.

### Statistical analyses

Data were analyzed using GraphPad Prism 5 software. The statistical tests were one-way or two-way ANOVA followed by Bonferroni's or Tukey posthoc analysis for multiple comparisons test, unpaired two-tailed Student’s t-test for analyzing the significance between two groups, and correlations with Pearson r test or with Spearman test for nonparametric analysis. All results were expressed as mean ± standard error of the mean (SEM), and *p* values < 0.05 were considered significant. All individual data with statistical analysis can be found in S1 Table.

## Results

### *App* gene expression is increased in the postnatal period in the Ts65Dn brain

**Fig 1:**
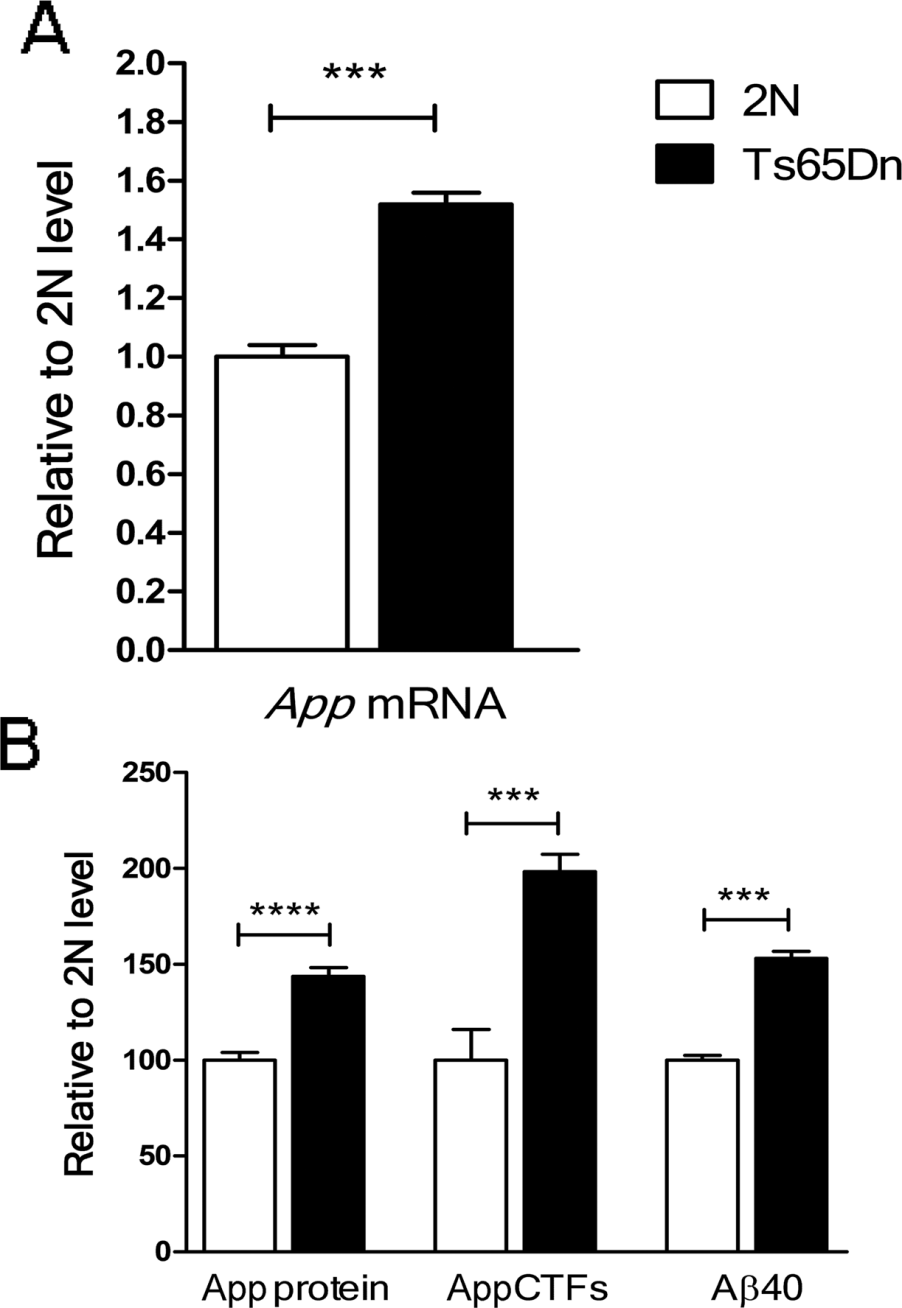
APP expression in the brain of postnatal Ts65Dn mice. (A) APP mRNA was significantly increased in the Ts65Dn brain relative to the 2N control (*p* = 0.006). The data are mean ± SD, n (pooled samples) = 6. (B) Full length APP, CTFs and Aβ40 were significantly increased in the Ts65Dn brain as compared to 2N controls (*p* = 0.00001, *p* = 0.002 and *p* = 0.002 respectively). The values are mean ± SEM: the number of samples (N for mice, n for pooled samples) was: full length APP: 2N, N = 5; Ts65Dn, N = 5. APP-CTF’s: 2N, N = 5; Ts65Dn, N = 5. Aβ 40: 2N, n = 3; Ts65Dn, n = 3. ***—*p* < 0.001, ****—*p* < 0.0001. Error bars, SEM. *p* values were calculated using two-tailed Student T test.

Because DS-01 presents the Aβ peptide as antigen, we asked at which time-point the increased App gene dose resulted in increased APP and its proteolytic products, including Aβ. Increases in mRNA, the full length APP protein, as well as its products, were present by postnatal day 21 (Fig 1A and 1B). The observed increases approximated gene dose. Despite the small number of analyzed samples, these data show increases in App mRNA, full length A, APP-CTF and Aβ40 as early as the end of the developmental period in Ts65Dn mice. These data complements earlier studies documenting changes of the same magnitude during adult life and advanced age [33]. Thus, vaccination against Aβ can be used to address the impact of APP products in young and old mice.

### Vaccination with DS-01 generated anti-mouse Aβ antibodies

**Fig 2:**
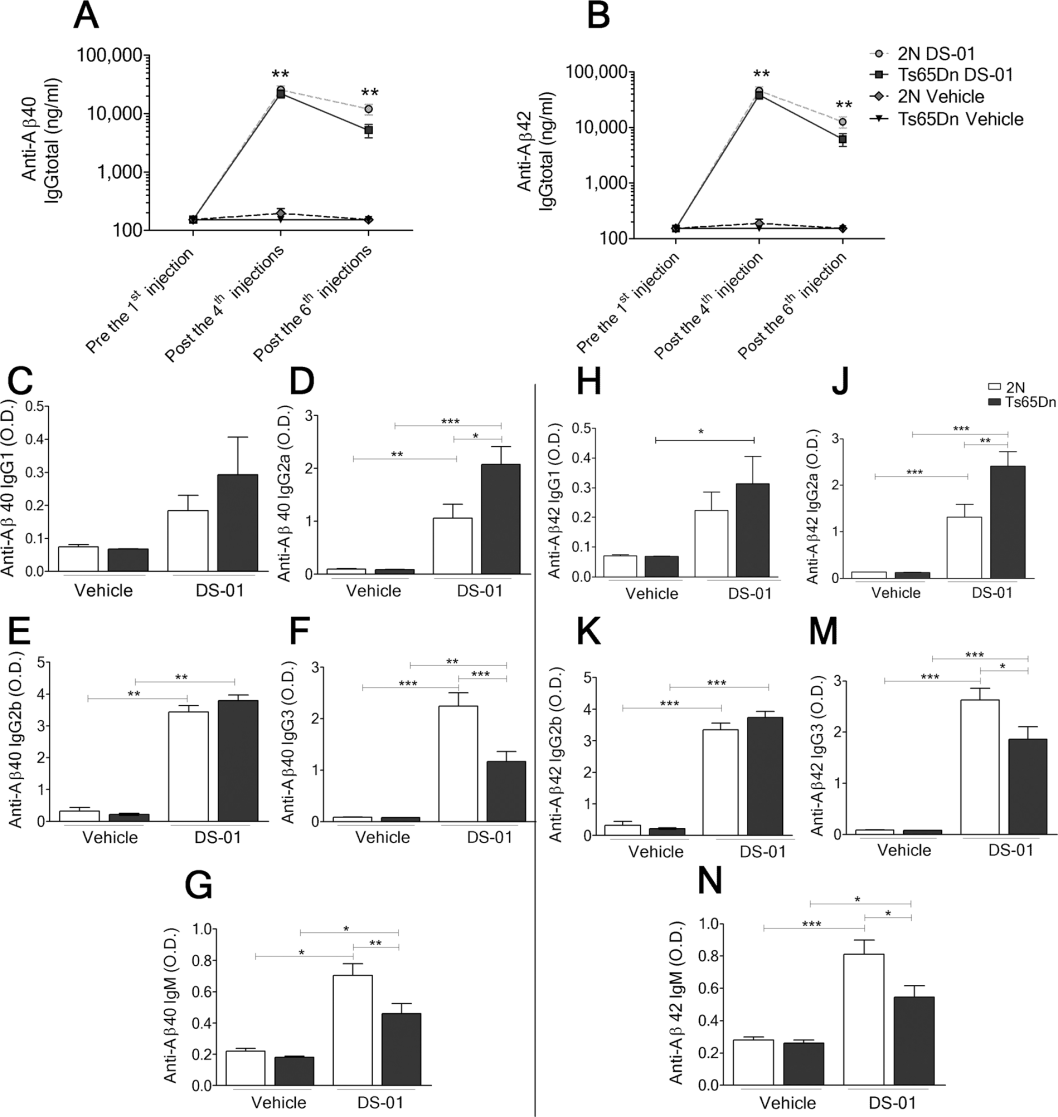
Anti-mouse Aβ antibody levels in 2N and Ts65Dn mice immunized with either vehicle or DS-01. (A) Anti-mouse-Aβ40 and (B) Aβ42 IgG titers were detected in plasma of DS-01 immunized mice following the 2^nd^ and the 4^th^ injection. Significant titers remained as late as 40 days after the 6^th^ injection. There was no significant difference between titers in 2N versus Ts65Dn mice. (C to F) Analysis of anti-mouse-Aβ40 IgG isotypes following the 4^th^ immunization. (G) Anti mouse Aβ40 IgM titers were lower in Ts65Dn mice. (H to M) Analysis of anti-mouse-Aβ42 IgG isotypes following the 4^th^ immunization. (N) Anti mouse Aβ42 IgM titers were lower in Ts65Dn mice. One-way ANOVA, Bonferroni's multiple comparison test *—*p* < 0.05; **—*p* < 0.01; ***—*p* < 0.001. Error bars, SEM. The number of mice was: 2N-vehicle/Ts65Dn-vehicle/2N-DS-01/Ts65Dn-DS-01 = 18/11/20/15.

DS-01 was prepared using an established liposome-technology, with tetrapalmitoylated mouse Aβ 1–15 peptide embedded into liposomes along with MPLA [35]. Six doses of either vehicle (liposome without the peptide) or DS-01 were administered to age-matched 2N and Ts65Dn mice. ELISA analysis of plasma after the 4^th^ dose, showed robust IgG titers against murine Aβ40 and Aβ42 in DS-01 immunized 2N and Ts65Dn mice (Fig 2A and 2B). Increased titers persisted for at least 40 days after the last immunization. No titers were detected in vehicle-immunized mice (Fig 2A and 2B).

Subclass analysis of anti-Aβ IgGs following the 4^th^ immunization showed similar IgG1 and IgG2b titers for 2N and Ts65Dn mice (Fig 2C and 2E). Ts65Dn mice showed significantly higher IgG2a titers and significantly lower IgG3 titers than 2N mice (Fig 2D and 2F). Anti-Aβ IgM titers were also lower in Ts65Dn mice (Fig 2G). Similar results for IgG subclasses and IgM were detected for Aβ42 (Fig 2H to 2N).

### Characterization of vaccine-induced antibodies

**Fig 3:**
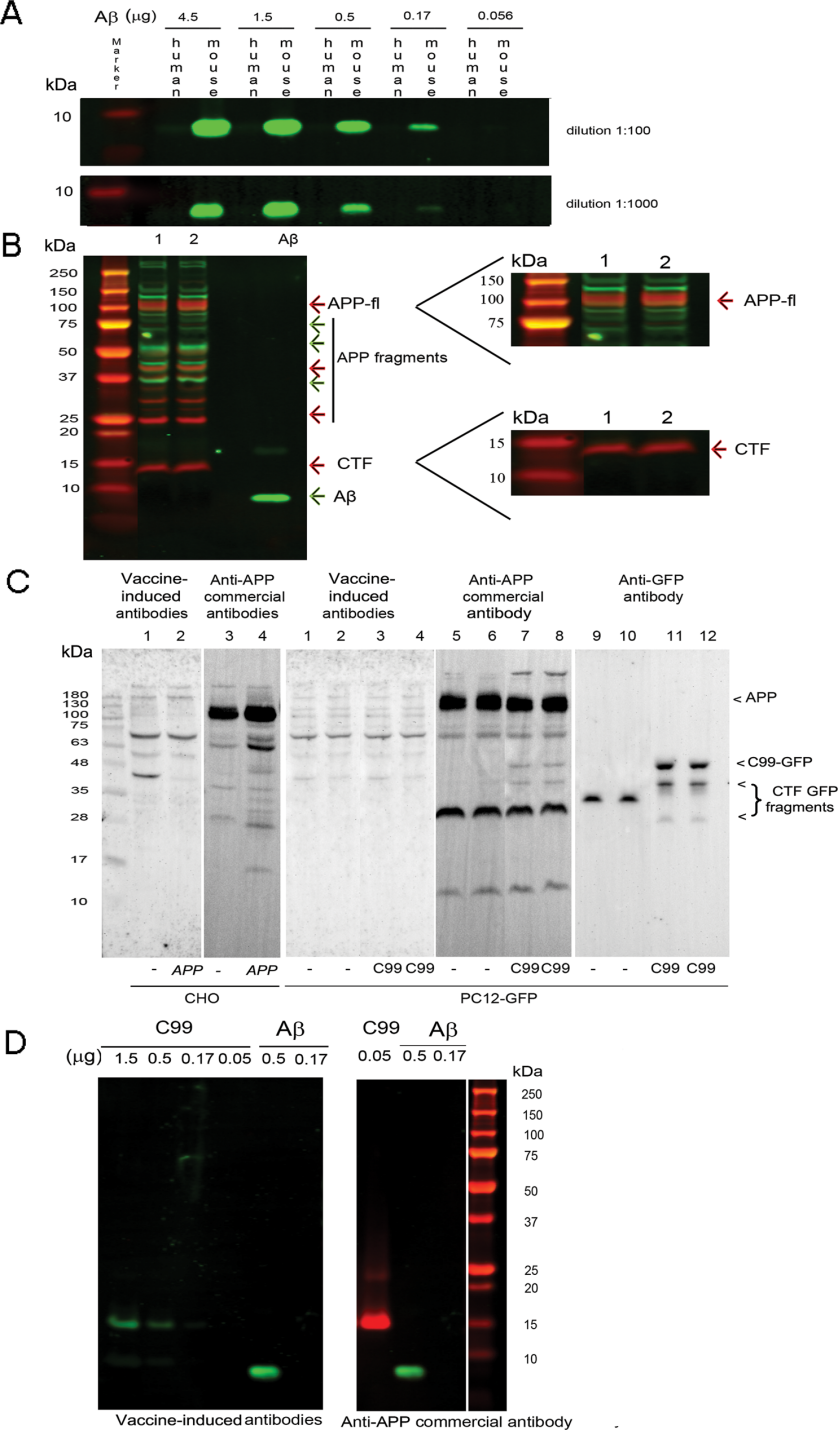
Characterization of vaccine-induced plasma immunoreactivity. (A) Assessment of immunoreactivity against human and mouse Aβ. Different quantities of mouse or human Aβ were blotted with dilutions of plasma (1:100 and 1:1000). The vaccine-induced antibodies were specific to mouse Aβ. (B) Western blots of two homogenates from Ts65Dn (lane 1) and 2N brains (lane 2) comparing vaccine-induced plasma (green signals) and a commercial anti-Aβ antibody to the C-terminus of APP (red signals). Only the commercial APP C-terminal antibody allowed the detection of APP and CTF. Unidentified bands were also detected using each of the antibodies, but no overlapping bands were observed, best appreciated in the right panel at higher magnification. The brain samples loaded were: vehicle-treated Ts65Dn (lane 1), vehicle-treated 2N (lane 2), synthetic mouse Aβ (lane 3). (C) Western blots of homogenates from CHO or PC12 cells using vaccine-induced plasma and a commercial anti-Aβ antibody. (Left panel) Lysates of wild type CHO cells (lanes 1 and 3), or CHO cells transfected with APP (lanes 2 and 4), were probed with plasma (1:1000) (lanes 1 and 2) or with the APP C-terminal antibody (1:1000) (lanes 3 and 4). (Right panel) The lysates of PC12 cells transfected with GFP alone were probed with plasma (lanes 1 and 2), with the APP C-terminal antibody (lanes 5 and 6) or with anti-GFP antibody (lanes 9 and 10). The lysates of PC12 cells expressing C99/GFP probed with plasma (lanes 3 and 4), with the APP C-terminal antibody (lanes 7 and 8), or with anti-GFP antibody (lanes 11 and 12). There was no cross-reactivity of vaccine-induced plasma with full length APP or CTFs. (D) Varying amounts of recombinant C99 were blotted with the vaccine-induced plasma (green bands) or with a commercial anti-APP antibody (red band). Vaccine-induced plasma demonstrated sensitivity at least 30-fold less than the APP C-terminal antibody.

To characterize vaccine-induced antibodies, we examined immunoreactivity against synthetic peptides and homogenates of mouse brain or cells expressing human APP. Fig 3A shows that, immunofluorescence signals were detected for synthetic mouse but not human Aβ. Fig 3B shows immunoblotting of brain homogenates from Ts65Dn (lane 1) and 2N mice (lane 2) with vaccine-induced plasma (green signal) and a commercial antibody specific for the C-terminus of APP (red signal). The vaccine-induced antibodies failed to detect full length APP or CTFs; no significant reactivity was detected at the migration position for these species (magnification, right panel, no overlap of green and red signals). With each antibody, additional bands were detected; their identity is unknown, but there was no overlap in staining between vaccine-induced plasma and the APP C-terminal antibody. As in panel A, vaccine-induced plasma was immunoreactive with mouse synthetic Aβ (lane 3). While we cannot rule out different affinities and/or epitopes for the commercial and vaccine-induced antibodies, we detected no vaccine-induced reactivity against full length mouse APP or its CTFs.

To explore further vaccine-induced immunoreactivity, we probed membranes bearing homogenates of cells that overexpress either human APP or C99. Fig 3C (left panel) reports results for CHO cells that were wild type or overexpressed APP. Overexpression of APP resulted in an increase in immunoreactivity at the position expected for full length APP (Fig 3C, left panel, lanes 3 versus 4). No significant immunoreactivity was detected at this position when probing with plasma in either wild type or APP overexpressing cells. To examine reactivity against C99, PC12 cells were transfected with a construct encoding a C99/GFP fusion protein or with GFP alone and homogenates were probed with the plasma or with the APP C-terminal antibody. Using the latter, bands at the positions expected for APP and the APP CTF, likely α-CTF C83, were detected in GFP-expressing cells (lanes 5, 6). A prominent 30 kDa band is undefined. Neither APP nor the CTF were detected with vaccine-induced plasma (lanes 1, 2). For PC12 cells expressing C99/GFP, the commercial antibody again detected APP and the CTF (lanes 7, 8). Note in addition, the presence of bands at approximately 48 and 35 kDa; the more slowly migrating band corresponds to that expected for C99/GFP fusion protein, while that at 35 kDa may represent a partially cleaved product of C99/GFP. These bands were absent when probing with the plasma (lanes 3, 4). Finally, antibodies to GFP detected the presence of a single band that corresponds to GFP in cells that expressed GFP alone (lanes 9, 10); in C99/GFP expressing cells 48 and 35 kDa bands (lanes 11, 12) were detected that were also seen with the APP antibody (lanes 7, 8). These data are further evidence against immunoreactivity of vaccine-induced plasma for APP or C99.

Next we asked if the plasma would detect recombinant C99. In Fig 3D, the blot was first probed with the plasma and then with the antibody to the APP C-terminus (plasma-green signal; APP C-terminal antibody-red signal). The plasma detected C99 under these conditions (left panel), but with a sensitivity that was at least 30-fold less than the APP C-terminal antibody (Fig 3D, compare signals for C99–1.5 μg in left panel versus 0.056 in right panel). We conclude there was no evidence for immunoreactivity for C99 or APP in mouse and human biologically produced samples and that vaccine-induced antibodies bound recombinant C99 much less strongly than the commercial anti-APP and anti-C99 antibodies at the same dilution.

### DS-01 vaccination had a trend to reduce Aβ42 and Aβ40 levels in the Ts65Dn brain

**Fig 4:**
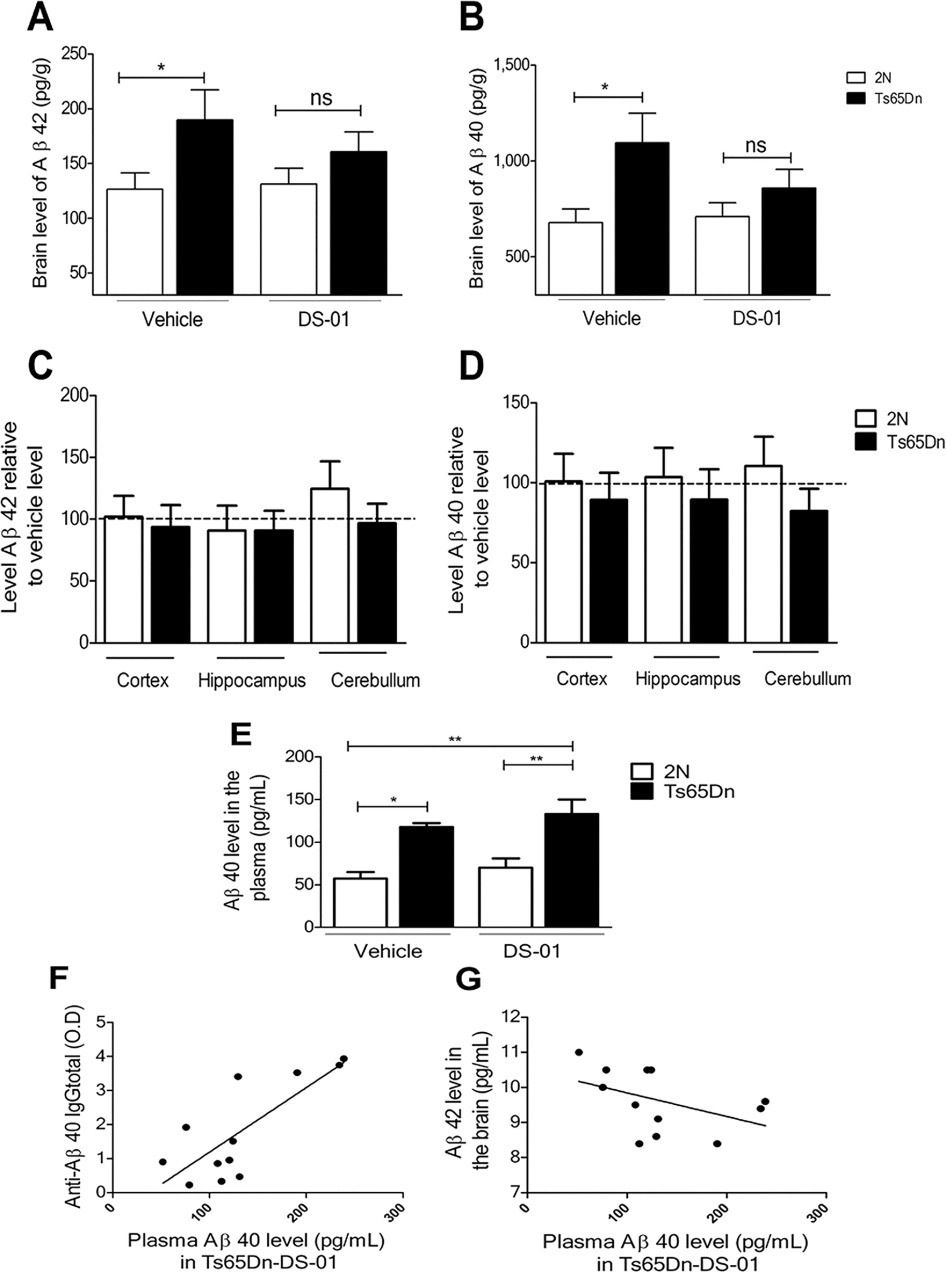
DS-01 immunization resulted in a trend to reduced levels of Aβ42 and Aβ40 in the Ts65Dn brain. (A) The levels of Aβ42 and (B) Aβ40 in brain samples from 2N and Ts65Dn mice. With respect to 2N vehicle-treated mice, Ts65Dn vehicle-treated mice showed significantly higher levels of Aβ42 and Aβ40 (two-tailed Student T test *p* = 0.031 and *p* = 0.007 respectively). In Ts65Dn immunized mice, Aβ42 and Aβ40 levels were not significantly different to those in 2N immunized mice (two-tailed Student T test *p* = 0.21 and *p* = 0.22). The number of mice used was as follows: 2N-vehicle/Ts65Dn-vehicle/2N-DS-01/Ts65Dn-DS-01 = 18/11/19/13. (C and D) Ratio of regional levels of Aβ42 and Aβ40 in 2N and Ts65Dn immunized mice relative to their corresponding vehicle controls. For Ts65Dn mice, but not 2N mice, there was a trend to lower Aβ levels following vaccination. This was most evident for Aβ40. The number of mice used was as follows: 2N-DS-01/Ts65Dn-DS-01 = 10/8. (E) Aβ40 in the plasma was increased in Ts65Dn mice (One-way ANOVA, Bonferroni's multiple comparison test *p* < 0.05); following immunization with DS-01, it was further increased, but the difference was not statistically significant (*p >* 0.05). The number of mice used was as follows: 2N-vehicle/Ts65Dn-vehicle/2N-DS-01/Ts65Dn-DS-01 = 7/10/8/12. (F) The level of anti-Aβ40 IgG titers correlated with the Aβ40 level in the plasma of Ts65Dn mice treated with the DS-01. (Pearson r correlation 0.6, *p* = 0.04). The number of mice used was: Ts65Dn-DS-01 = 12. (G) An inverse correlation was found between the level of Aβ42 in the brain and in the plasma (Pearson r correlation -0.5 *p* = 0.012). The number of mice used was: Ts65Dn-DS-01 = 12. ns- non-significant, *—*p* < 0.05, **—*p* < 0.01. Error bars, SEM.

ELISA was used to examine DS-01 effects on brain Aβ levels using extracts from the combined hippocampus, cortex and cerebellum. In vehicle-treated mice, Aβ42 (Fig 4A) and Aβ40 (Fig 4B), Aβ levels were increased in TS65Dn mice with respect to 2N mice. Relative to vehicle, there was a decrease in the levels of Aβ in DS-01-treated Ts65Dn mice, but the reductions failed to reach statistical significance. Nevertheless, Aβ42 and Aβ40 levels in DS-01-treated Ts65Dn mice were no longer statistically significantly different from those in 2N mice. Fig 4C and 4D plots the levels in individual regions for Aβ in DS-01- versus vehicle-treated mice; no significant decrease was detected for Aβ42 or Aβ40, but Ts65Dn regions showed a consistent trend toward decreased Aβ40. We conclude that vaccination had a modest, albeit statistically insignificant, effect in reducing Aβ levels in the Ts65Dn brain.

Aβ level was also measured in plasma (Fig 4E); only Aβ40 was detected. Increased Aβ40 in Ts65Dn versus 2N mice were present in both vehicle-treated and DS-01-treated mice. Following DS-01 treatment, a small but non-significant increase of Aβ40 was registered in the plasma of Ts65Dn mice. To explore further the disposition of Aβ in vaccinated mice, we tested for possible correlations between plasma Aβ and antibody levels. Plasma Aβ40 levels correlated positively with plasma anti-Aβ IgG (Fig 4F), suggesting that vaccination enhanced transit of Aβ from brain to blood. In addition, in DS-01-treated Ts65Dn mice, there was a significant negative correlation between brain Aβ42 and plasma Aβ40 (Fig 4G), a finding consistent with communication between brain and plasma pools in these mice.

**Fig 5:**
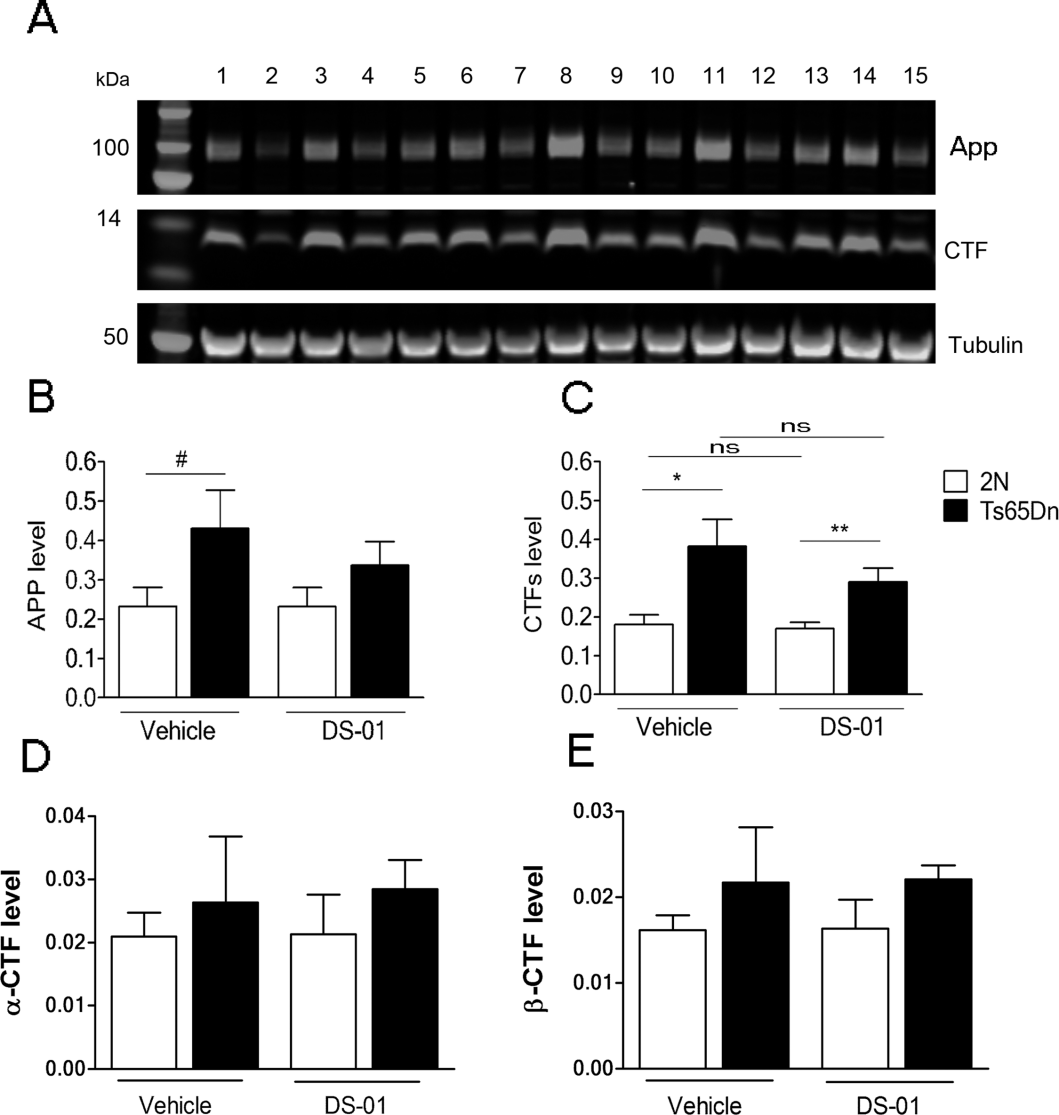
Vaccination had no significant effect on the levels of APP or CTFs. (A) Western blot showing bands for APP and CTFs in brain samples from 2N and Ts65Dn mice. Tubulin was used as internal reference. The lanes are: 2N-vehicle (2, 6, 10, 13); Ts65Dn-vehicle (4, 8); 2N-DS-01 (1, 5, 9, 12, 15); Ts65Dn-DS-01 (3, 7, 11, 14). (B) Quantification of APP showed a higher level in Ts65Dn mice (although here it reached only borderline significance, *p* = 0.07). Following treatment with DS-01, no significant difference was observed in APP relative to the vehicle for either genotype (2N, vehicle vs DS-01, *p* = 0.9; Ts65Dn; vehicle vs DS-01, *p* = 0.4). (C) Quantitation of CTFs revealed significantly higher levels in T65Dn brains in both vehicle-treated and vaccine-treated mice (2N vehicle vs Ts65Dn vehicle, *p* = 0.01; 2N DS-01 vs Ts65Dn DS-01, *p* = 0.008). Following DS-01 treatment, no significant difference was observed in CTFs (2N, vehicle vs DS-01, *p* = 0.7; Ts65Dn; vehicle vs DS-01, *p* = 0.2). The number of mice used for APP and CTFs was: 2N- vehicle/Ts65Dn- vehicle/2N-DS-01/Ts65Dn-DS-01 = 7/5/8/8. (D) Quantification of α-CTF and (E) β-CTF levels in vehicle-treated and immunized mice. There was no significant effect of vaccine-treatment (α-CTFs: 2N, vehicle vs DS-01 *p* = 0.9; Ts65Dn, vehicle vs DS-01 *p =* 0.8.; β-CTF: 2N, vehicle vs DS-01 *p* = 0.9; Ts65Dn, vehicle vs DS-01 *p =* 0.9). The number of mice used was: 2N- vehicle/Ts65Dn- vehicle/2N-DS-01/Ts65Dn-DS-01 = 4/5/5/7. Error bars, SEM. All statistical analyses were performed using two-tailed Student T test #, *p* = 0.07, ns- non-significant, *—*p* < 0.05, **—*p* < 0.01.

To ask if vaccination impacted APP or CTFs, we measured their levels in vehicle- and DS-01-treated mice The levels of APP, CTFs, a-CTF and b-CTF were measured by immunoblots and Fig 5A–5E demonstrates no significant effect of vaccination on APP or its CTFs in either Ts65Dn or 2N mice.

### Improved memory in DS-01 vaccinated Ts65Dn mice

**Fig 6:**
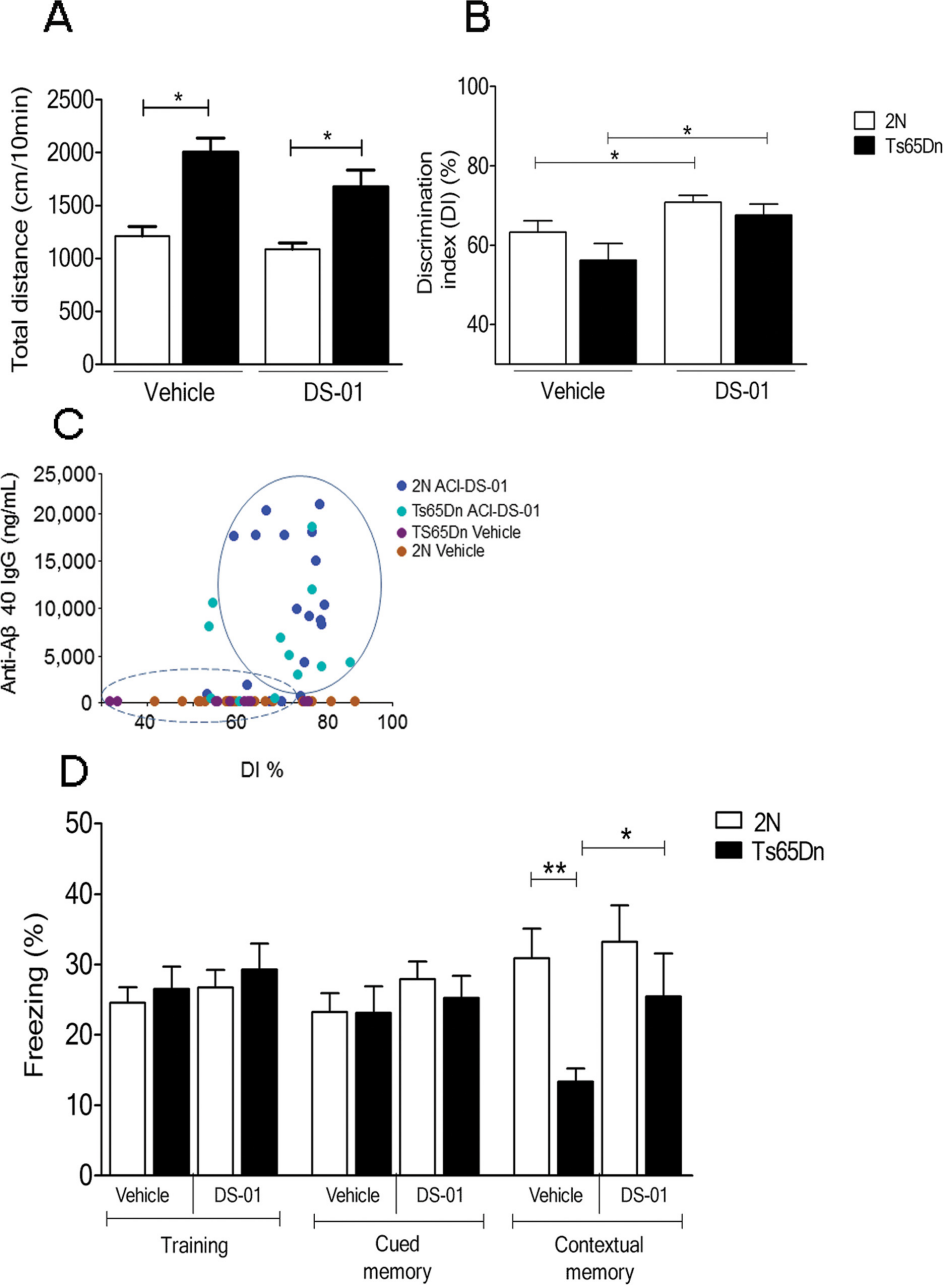
Behavioral evaluation and memory function following DS-01 immunization. (A) The difference in spontaneous locomotor activity between 2N and Ts65Dn mice was unaffected by immunization. (B) In comparison to mice treated with vehicle, both 2N and Ts65Dn immunized with DS-01 showed significantly enhanced discrimination index (DI) in the novel object recognition test (two-tailed Student T test, *p* = 0.03). The number of mice used was as follows: 2N- vehicle/Ts65Dn- vehicle/2N-DS-01/Ts65Dn-DS-01 = 18/11/20/13. (C) Positive correlation between the level of anti-Aβ40 IgG and the DI (Spearman r correlation 0.4, *p* = 0.002). Mice having no titers and spreading over the entire range of DI are those immunized with vehicle. The two top performing (highest DI%) are vehicle-treated 2N mice and the two worth performing (lowest DI%) are vehicle-treated Ts65Dn mice. Data from both 2N and Ts65Dn mice immunized with DS-01 are spread in a cloud (solid circle) above DI of 70% while the majority of vehicle-immunized mice had a lower DI value (dashed circle). (D) In the fear conditioning test, during the contextual session, vehicle-treated Ts65Dn mice showed significantly less freezing versus 2N vehicle-treated mice (two-tailed Student T test, *p* = 0.004). In vaccinated Ts65Dn mice, freezing was significantly different from vehicle-treated Ts65Dn (two-tailed Student T test *p* = 0.05) and not significantly different from that in 2N vaccinated mice (two-tailed Student T test *p* = 0.3). *—*p* < 0.05, **—*p* < 0.01; Error bars, SEM. The number of mice used was as follows: 2N- vehicle/Ts65Dn- vehicle/2N-DS-01/Ts65Dn-DS-01 = 18/11/20/12.

Mouse models of DS exhibit behavioral phenotypes that distinguish them from 2N mice [32,37]. Ts65Dn mice show abnormalities in some tests of hippocampally-mediated memory. To investigate an effect of the DS-01 vaccine on cognition, tests were conducted two weeks after the last immunization in this order: locomotor activity, object recognition, and fear conditioning. Vehicle-treated Ts65Dn mice in comparison to 2N mice showed, as reported [31] higher spontaneous locomotion that was not altered by DS-01 treatment (Fig 6A). Recognition memory was measured in the novel object recognition task. Ts65Dn mice showed a lower discrimination index (DI) by 11%–i.e. spent less time exploring a novel object than 2N mice. DS-01-treated Ts65Dn mice showed a significantly higher DI, providing evidence for improvement in this test of memory (Fig 6B). Interestingly, a similar improvement was observed in 2N mice after DS-01 treatment, but the effect was smaller than for Ts65Dn mice. Of note, in the novel object recognition task, despite a low correlation coefficient (Spearman test r = 0.4, *p* = 0.002), the DI score correlated positively with anti-Aβ IgG titers (Fig 6C).

The fear conditioning test examines contextual memory. Similar levels of freezing were observed in all groups during training and cued memory sessions (Fig 6D). During the contextual memory session, vehicle-treated Ts65Dn mice showed significantly lower freezing than 2N mice; this is interpreted as an inability to recognize the context in which fear was induced. In contrast, DS-01-immunized Ts65Dn mice demonstrated a marked (~90%) increase in freezing as compared to the vehicle-treated Ts65Dn mice; freezing reached the level in 2N mice. Vaccine treatment thus reduced the defect in contextual fear memory in Ts65Dn mice and improved behaviors linked to hippocampally-mediated cognition.

### Vaccination of Ts65Dn mice prevented atrophy of medial septum cholinergic neurons

**Fig 7:**
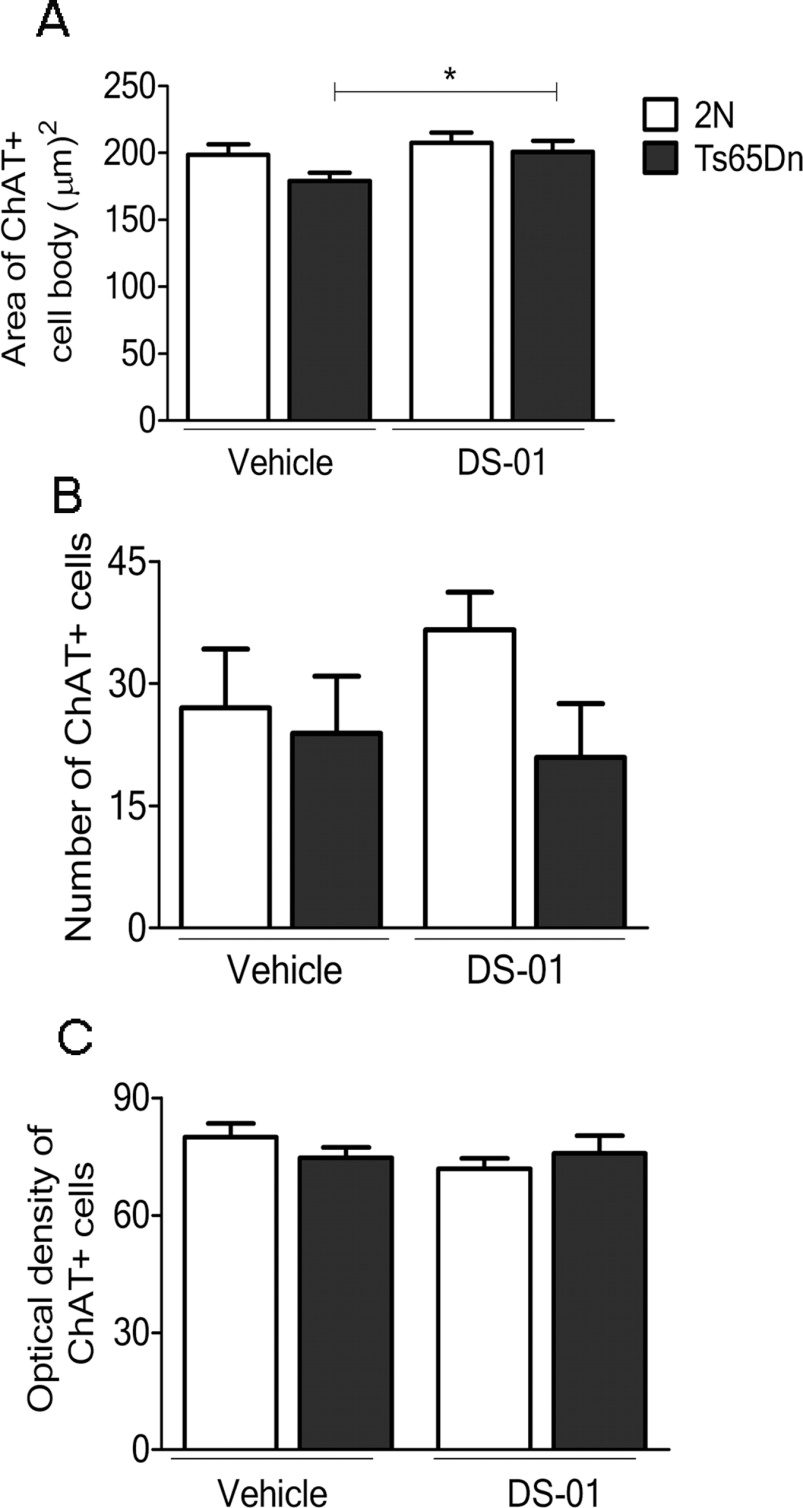
Immunization with DS-01 prevented the atrophy of cholinergic neurons. (A) The area of ChAT+ cell bodies was significantly larger in Ts65Dn-DS-01 relative to Ts65Dn-vehicle treated mice (*p* = 0.03). (B) Number and **c** optical density of ChAT+ cells in medial septum were similar in DS-01-treated and vehicle-treated 2N and Ts65Dn mice. Two-tailed Student T test, *—*p* < 0.05. Error bars, SEM. The number of mice used was as follows: 2N- vehicle/Ts65Dn- vehicle/2N-DS-01/Ts65Dn-DS-01 = 4/4/4/4.

To ask if vaccine treatment impacted neurodegeneration, we analyzed cholinergic neurons in medial septum, as identified by immunohistochemical staining for choline acetyltransferase (ChAT). The area of ChAT-positive (ChAT+) cell bodies was significantly larger by ~10% in DS-01 versus-vehicle-treated Ts65Dn mice; following treatment with DS-01, cell body area in Ts65Dn mice was equivalent to that in DS-01-treated 2N mice (Fig 7A). Comparing DS-01 and vehicle-treated 2N and Ts65Dn mice, there was no significant change in the number of ChAT+ cells or in the optical density of staining in individual ChAT+ cells (Fig 7B and 7C). Therefore, the increase in size of ChAT+ cells was not due to increased immunostaining for ChAT. Since the size of these neurons is normal at the age vaccine treatment was initiated [48], we conclude that DS-01 vaccination prevented atrophy.

### The DS-01 vaccine did not result in inflammation, cellular infiltration, or hemorrhage

To assess the possible impact of immunization on the overall health of mice, we measured body and brain weight. The body weights of Ts65Dn mice were significantly lower relatively to 2N and the difference between them were unaffected by vaccination (2N-vehicle 43.8 g ± 4.9, Ts65Dn-vehicle 36.3 g ± 3.2, 2N-DS-01, 45.6 g ± 5.0, Ts65Dn-DS-01 37.5 g ± 4.1. Two-tailed Student t test 2N, vehicle vs DS-01 *p* > 0.001. Ts65Dn: vehicle vs DS-01 *p* > 0.001). There was no effect of immunization on brain weight in 2N or Ts65Dn mice (2N-vehicle 0.43 g ± 0.02, Ts65Dn-vehicle 0.44 g ± 0.02, 2N-DS-01 0.42 g ± 0.02, Ts65Dn-DS-01 0.043 g ± 0.01. Two-tailed Student t test 2N, vehicle vs DS-01 p > 0.001. Ts65Dn: vehicle vs DS-01 p > 0.001). Therefore, DS-01 treatment altered neither body weight nor brain weight in 2N or Ts65Dn mice. In addition, parameters of general health (i.e. physical appearance, food and water consumption) were not affected (data not shown).

**Fig 8:**
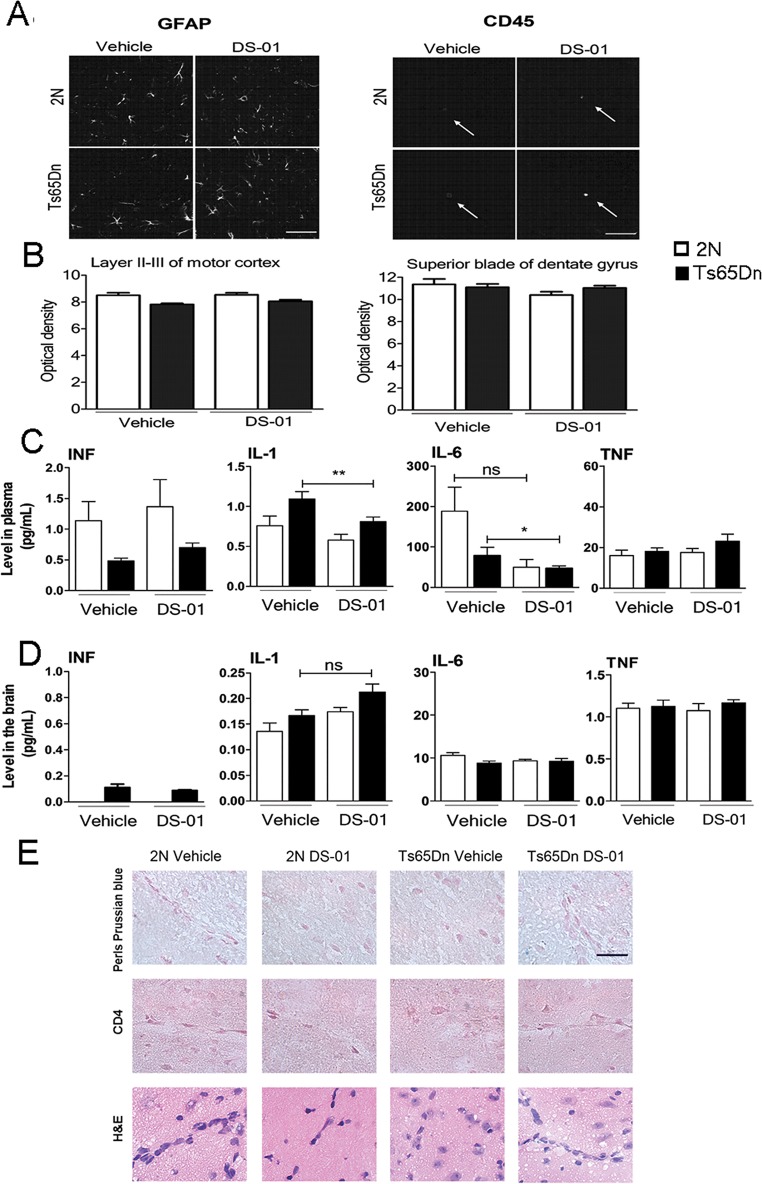
Measures of inflammatory markers following immunization with either vehicle or DS-01. (A) Confocal images of GFAP (left) and CD45 immunoreactivity (right) in vehicle- and DS-01 treated 2N and Ts65Dn mice. Arrows point individual CD45-positive microglial cells. Images are from cortex (Scale bars = 100μm). (B) Quantification of GFAP immunoreactive optical density revealed no difference between treatment groups in either layers II-II motor cortex (left) or superior blade of dentate gyrus (right). Error bars, SEM. The number of mice used was as follows: 2N- vehicle/Ts65Dn- vehicle/2N-DS-01/Ts65Dn-DS-01 = 4/4/4/4. (C and D) There was little if any effect of vaccination on the levels of IFN and TNF in the plasma and in brain extracts from 2N and Ts65Dn mice. IL-1 and IL-6 decreased following immunization in the plasma of Ts65Dn mice treated with the DS-01 vaccine (One-way ANOVA, Bonferroni's multiple comparison test **—*p* < 0.01 and *—*p* < 0.05 respectively). Error bars, SEM. The number of mice used was as follows: 2N-vehicle/Ts65Dn-vehicle/2N-DS-01/Ts65Dn-DS-01 = 7/10/8/12. (E) Staining of cortical sections with Perls Prussian blue, immunostaining with anti-CD4 antibody, and H&E revealed the absence of positive staining in all experimental groups. These results are evidence against lymphocytic infiltration or microhemorrhage in vaccinated mice. The number of mice used was as follows: 2N- vehicle/Ts65Dn- vehicle/2N-DS-01/Ts65Dn-DS-01 = 3/5/5/5.

To determine if inflammation attended vaccination, we evaluated astroglial activation by examining glial fibrillary acidic protein (GFAP) and microglial activation, by examining CD45. Immunostaining for GFAP and CD45-positive cells (Fig 8A) showed no significant differences between 2N or Ts65Dn mice, vehicle- and DS-01 treated mice in both cortex and hippocampus regions (Fig 8B). In addition, pro-inflammatory markers were examined (Fig 8C and 8D). In plasma, immunization with DS-01 had no significant effect on the levels of IFN or TNF in either 2N or Ts65Dn mice. Interestingly, following immunization, IL-1 and IL-6 levels were reduced significantly in the Ts65Dn group (Fig 8C). In brain extracts, IFN was not detected in any groups and DS-01 immunization had no significant effect on Il-6 or TNF levels (Fig 8D). DS-01 immunization did result in an increase in IL-1 levels in the brain of both the 2N and the Ts65Dn mice, but the changes did not reach statistical significance. There was no evidence of hemorrhage in DS-01-treated mice, as exemplified using an iron stain (Fig 8E), nor was there evidence for lymphocytic infiltration as measured by immunostaining for CD4 cells. We conclude that vaccination was without evident changes in brain weight, brain injury or inflammatory markers.

## Discussion

We demonstrate that the DS-01 vaccine was potent, safe and effective in a mouse model of DS. The findings are the first to report on immunization against Aβ in a model of DS. Vaccinated Ts65Dn brains demonstrated consistent trends for reductions in Aβ42 and Aβ40 resulting in values that were no longer statistically different with respect to 2N values. Most importantly, vaccination acted to prevent cholinergic neuron atrophy and improved hippocampal-dependent memory function. The findings support the view that targeting Aβ via immunization is a rational strategy for treating AD-like symptoms in people with DS.

An important question is whether immunization can be used to reverse and/or prevent existing AD-like relevant phenotypes in DS. It is noteworthy that herein immunization was initiated in the presence of increased APP gene expression, as reflected in increased levels of the full length protein and its products, including Aβ42 and Aβ40. Previously, the efficiency of anti-Aβ vaccine to reduce Aβ level has been shown in euploid mice transgenic for a mutant form of APP [49]. The novelty of our work consisted in using a mouse model carrying un-mutated APP gene and having a close genetic background to DS meaning the murine chromosome 16 and a part of chromosome 17 in triplicate rather than over-expressing one human gene. Vaccine effects were registered in tests of both cognition and cellular degeneration. While additional studies will be needed to more fully define vaccination effects, the data encourage the view that vaccination impacts both established and developing DS-relevant neurological phenotypes. The improvement in cognitive function is rationally due to restoration of function since the examined measures are early in onset, present by 3 months of age–i.e. months before anti-Aβ titers would have been induced. In contrast, atrophy of basal forebrain cholinergic neurons, a population markedly affected in AD, DS and mouse models of DS [33], occurs after 6 months of age–i.e. after the onset of vaccination [48]. The vaccine effect on cell size is consistent with preventing this aspect of neurodegeneration. In addition, DS-relevant deficits in learning and memory might be not reproducible [50]. However, our data generated in two behavioral tasks, reinforced the hypothesis that vaccination can be considered among the efficient drugs to rescue learning and memory. Taken together, the data suggest that vaccine treatment can act to reverse as well as prevent disease-related phenotypes in the DS mouse model.

The ability to reverse behavioral changes supports a model of pathogenesis in which toxic species are envisioned to serve as ‘drivers’ of changes in neuronal structure and function [51]. The ‘driver hypothesis’ postulates that effectively engaging the target at any time will serve to reduce or even reverse injury. It is distinguished from the ‘trigger’ and ‘threshold’ hypotheses; they state that damage is essentially irretrievable once the initial toxic species appears to ‘trigger’ injury or reaches a certain ‘threshold’ at which injury ensues. Vaccine-mediated prevention of atrophy of basal forebrain cholinergic neurons does not distinguish between the hypotheses but does point to the ability to intercept this aspect of APP-linked pathogenesis. Whether these hypotheses apply to DS is unknown, but our findings suggest that they can be explored in mouse models of DS.

The antibody titers induced by the vaccine against Aβ were robust and persistent and Ts65Dn mice developed IgG titers as high as in 2N mice. This suggests that vaccine treatment may be able to overcome the impaired adaptive immune response to Aβ reported in people with DS [52]. Nevertheless, the observed lower IgM levels might indicate differences between Ts65Dn and 2N mice in immune reactivity that modify vaccine responsiveness [53,54]. It will be important to carefully address the possibility that the response to an Aβ–targeted vaccine may differ between the mouse model and people with DS. The mechanism by which the vaccine interacts with brain Aβ is yet to be defined. Further studies will be required to explore possible antibody-mediated brain to blood efflux as suggested by the correlation between increased plasma Aβ40 and increased anti-Aβ IgG in the Ts65Dn-DS-01 mouse.

To define the immunoreactivity induced by immunization we examined both biologically-produced sources of mouse and human APP, recombinant C99 and synthetic mouse and human Aβ. Reactivity was directed at mouse Aβ with little or no evidence of binding to APP or C99 in biological preparations and weak reactivity against recombinant C99. Thus, vaccine-induced immunoreactivity appears to have acted through targeting Aβ.

A crucial point concerns the safety of the vaccine. Our results raised no concerns for general health, brain inflammation or injury in the Ts65n mouse. There was no evident activation of astrocytes or microglia, lymphocytic infiltration or untoward findings in studies of cytokines in brain or in blood. Almost a decade ago, the first trial of anti-Aβ42 immune treatment in AD patient showed promising efficacy, with a slower rate of cognitive decline, [21,55] however 6% of treated patients developed meningoencephalitis due to Aβ42 specific CD4+ T-cell infiltration in brain [56,57]. Efforts to refine Aβ vaccines have resulted in reduced side effects [58] and several anti-Aβ vaccines have been in clinical trials [59]. Our findings encourage the view that a vaccine of the type used herein will be safe in people with DS, but underlying differences in immune function in DS mandates careful attention to this very important issue.

Immunotherapy has not yet been considered for treating cognitive impairment in people with DS. On the basis of our findings, the use of a vaccine against Aβ in DS may prove effective decades before the onset of full blown neuropathology and dementia. If Aβ vaccination can be shown to prevent AD-like related phenotypes in people with DS, this could provide unique insights and guidance for other clinical trials.

The development of an Aβ vaccine for clinical trials in DS would necessarily be preceded by a thorough safety evaluation and conducted with the proviso that subjects, and their caregivers or family members, were fully apprised of the risks and possible benefits of trial participation. Importantly, participants and caregivers must be informed that anti-Aβ immunotherapy targets age-related changes in brain function to effect treatment but not cure for those with DS.

## Supporting Information

S1 Table (XLSX)

## Abbreviations

Aβ: β-amyloid
AD: Alzheimer’s disease
APP: amyloid precursor protein
ChAT: choline acetyltransferase
DS: Down syndrome
GFAP: glial fibrillary acidic protein
IgG: Immunoglobulin
GAPDH: glyceraldehyde-3-phosphate dehydrogenase
MPLA: monophosphoryl lipid A
Pal1-15: tetrapalmitoylated mouse Aβ1-15-peptide
GFP: green fluorescent protein
CTF: C-terminal fragments
IFN: interferon-gamma
TNF: tumor necrosis factor
IL: interleukin
