# Correction: An Anti-β-Amyloid vaccine for treating cognitive deficits in a mouse model of down syndrome

**DOI:** 10.1371/journal.pone.0324280

**Published:** 2025-05-12

**Authors:** Pavel V. Belichenko, Rime Madani, Lorianne Rey-Bellet, Maria Pihlgren, Ann Becker, Adeline Plassard, Stephanie Vuillermot, Valérie Giriens, Rachel L. Nosheny, Alexander M. Kleschevnikov, Janice S. Valletta, Sara K. S. Bengtsson, Gordon R. Linke, Michael T. Maloney, David T. Hickman, Pedro Reis, Anne Granet, Dorin Mlaki, Maria Pilar Lopez-Deber, Long Do, Nishant Singhal, Eliezer Masliah, Matthew L. Pearn, Andrea Pfeifer, Andreas Muhs, William C. Mobley

This notice is issued to correct image preparation errors with Figs 3 and 8. During the preparation of the western blot results presented in Fig 3 of the original article [1], the underlying gels and blots were spliced to remove non-pertinent lanes, but the splice lines were not clearly marked in the published figure. In addition, during the splicing of the blots, incorrect lanes were selected during the preparation of Figs 3B and 3C. The updated Fig 3 below presents the corrected panels. The original blots underlying the original and corrected Fig 3 results are provided in S1 File.

**Fig 3:**
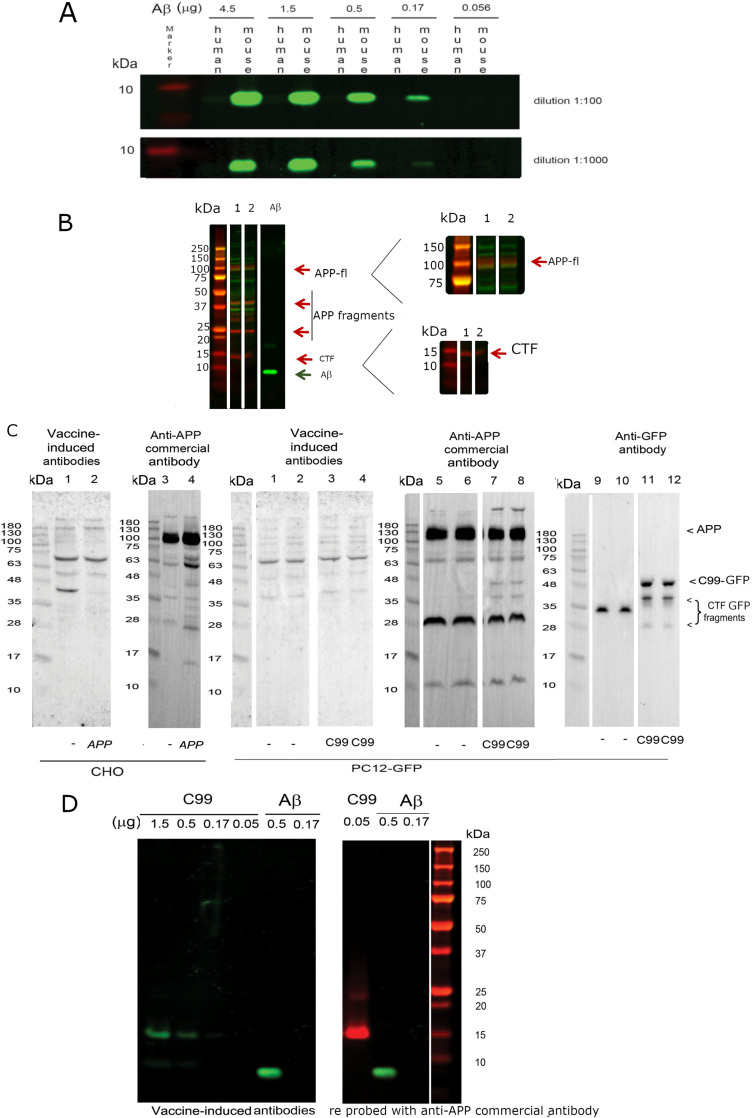
Characterization of vaccine-induced plasma immunoreactivity. (A) Assessment of immunoreactivity against human and mouse Aβ. Different quantities of mouse or human Aβ were blotted with dilutions of plasma (1:100 and 1:1000). The vaccine-induced antibodies were specific to mouse Aβ. (B) Western blots of two homogenates from Ts65Dn (lane 1) and 2N brains (lane 2) comparing vaccine-induced plasma (green signals) and a commercial anti-Aβ antibody to the C-terminus of APP (red signals). Only the commercial APP C-terminal antibody allowed the detection of APP and CTF. Unidentified bands were also detected using each of the antibodies, but no overlapping bands were observed, best appreciated in the right panel at higher magnification. The brain samples loaded were: vehicle-treated Ts65Dn (lane 1), vehicle-treated 2N (lane 2), synthetic mouse Aβ (lane 3). (C) Western blots of homogenates from CHO or PC12 cells using vaccine-induced plasma and a commercial anti-Aβ antibody. (Left panel) Lysates of wild type CHO cells (lanes 1 and 3), or CHO cells transfected with APP (lanes 2 and 4), were probed with plasma (1:1000) (lanes 1 and 2) or with the APP C-terminal antibody (1:1000) (lanes 3 and 4). (Right panel) The lysates of PC12 cells transfected with GFP alone were probed with plasma (lanes 1 and 2), with the APP C-terminal antibody (lanes 5 and 6) or with anti-GFP antibody (lanes 9 and 10). The lysates of PC12 cells expressing C99/GFP probed with plasma (lanes 3 and 4), with the APP C-terminal antibody (lanes 7 and 8), or with anti-GFP antibody (lanes 11 and 12). There was no cross-reactivity of vaccine-induced plasma with full length APP or CTFs. (D) Varying amounts of recombinant C99 were blotted with the vaccine-induced plasma (green bands) or with a commercial anti-APP antibody (red band). Vaccine-induced plasma demonstrated sensitivity at least 30-fold less than the APP C-terminal antibody.

**Fig 8:**
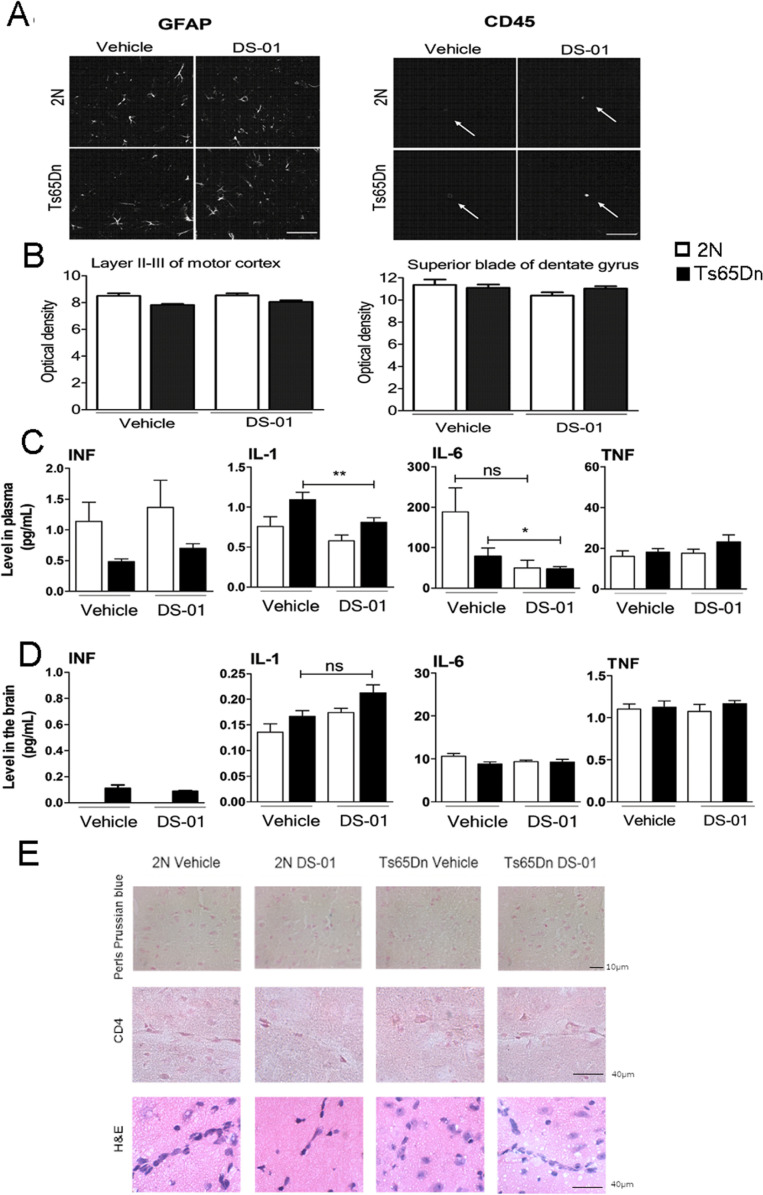
Measures of inflammatory markers following immunization with either vehicle or DS-01. (A) Confocal images of GFAP (left) and CD45 immunoreactivity (right) in vehicle- and DS-01 treated 2N and Ts65Dn mice. Arrows point individual CD45-positive microglial cells. Images are from cortex (Scale bars = 100μm). (B) Quantification of GFAP immunoreactive optical density revealed no difference between treatment groups in either layers II-III motor cortex (left) or superior blade of dentate gyrus (right). Error bars, SEM. The number of mice used was as follows: 2N- vehicle/Ts65Dn- vehicle/2N-DS-01/Ts65Dn-DS-01 = 4/4/4/4. (C and D) There was little if any effect of vaccination on the levels of IFN and TNF in the plasma and in brain extracts from 2N and Ts65Dn mice. IL-1 and IL-6 decreased following immunization in the plasma of Ts65Dn mice treated with the DS-01 vaccine (One-way ANOVA, Bonferroni’s multiple comparison test **—*p* < 0.01 and *—*p* < 0.05 respectively). Error bars, SEM. The number of mice used was as follows: 2N-vehicle/Ts65Dn-vehicle/2N-DS-01/Ts65Dn-DS-01 = 7/10/8/12. (E) Staining of cortical sections with Perls Prussian blue, immunostaining with anti-CD4 antibody, and H&E revealed the absence of positive staining in all experimental groups. These results are evidence against lymphocytic infiltration or microhemorrhage in vaccinated mice. The number of mice used was as follows: 2N- vehicle/Ts65Dn- vehicle/2N-DS-01/Ts65Dn-DS-01 = 3/5/5/5.

Furthermore, an incorrect panel was selected for the published Fig 8E Ts65Dn Vehicle results. The original data underlying the Perls Prussian blue results are no longer available at a consistent magnification of the panels published in the figure. The updated Fig 8 provided with this notice includes replacement panels obtained from the original Perls Prussian blue experiments at a lower magnification. The available image data underlying both the Fig 8 panels published in [1] and the updated Fig 8 results are provided in S2 File. The legend for Fig 8 has been updated to correct the typographical error “layer II-II” which should read “layer II-III” instead.

The S1 Table provided with [1] reports aggregate data, as opposed to individual-level underlying data. The S3 File available with this notice provides the individual-level data underlying the published results. The authors note that they were unable to locate the complete set of individual-level data underlying Fig 1, but they have provided the available raw PCR data underlying Fig 1A (S4 File).

## Supporting information

S1 File Original gels and blots underlying results presented in Figs 3 and 5. (ZIP)

S2 File Available image data underlying Fig 8E. (ZIP)

S3 File Individual-level data underlying published results in Figs 1–8. (XLSX)

S4 File Raw PCR data underlying Fig 1A results. (XLSX)
