# Supplementary figures and images for: Correction: An Anti-β-Amyloid vaccine for treating cognitive deficits in a mouse model of down syndrome
Source: PLoS One. 2025 May 12;20(5):e0324280. doi: 10.1371/journal.pone.0324280 (PMC12068576; doi:10.1371/journal.pone.0324280)

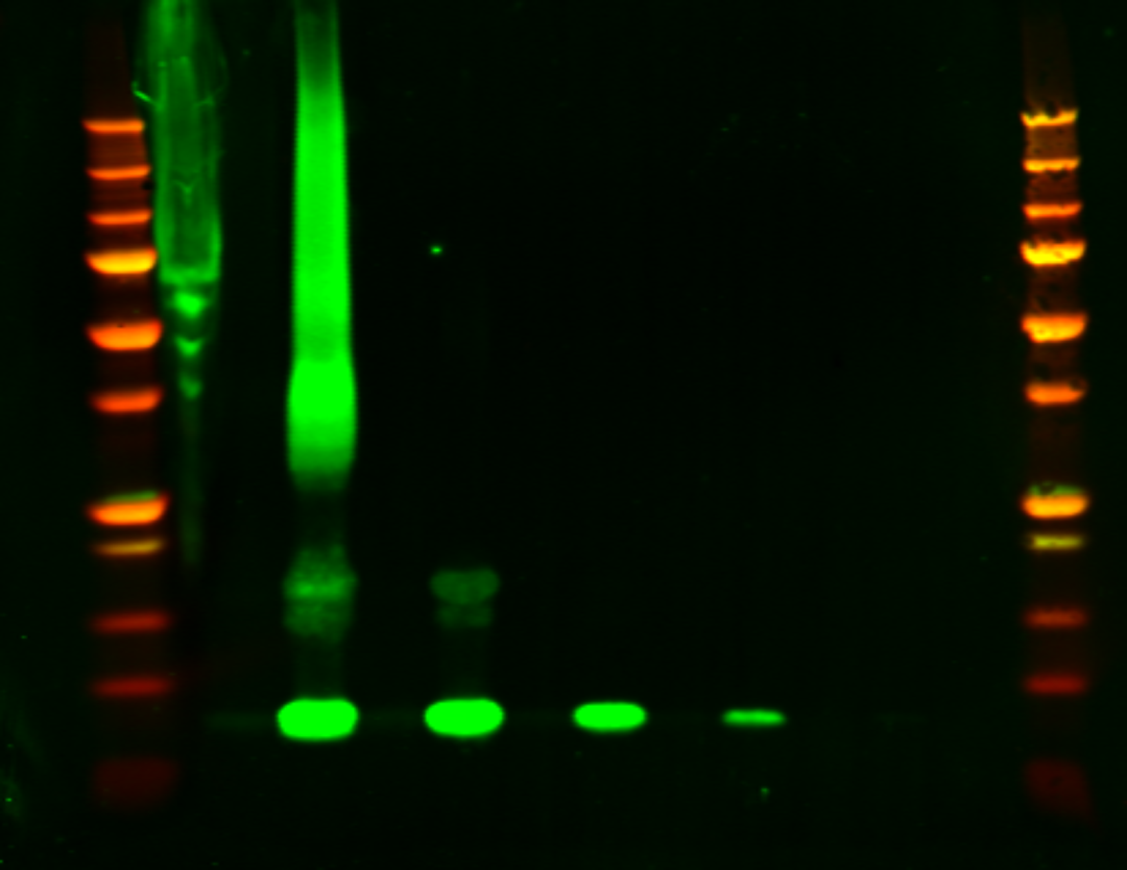

Supplement: S1 File — (ZIP) [file pone.0324280.s001.zip › S1 File/Figure 3 A 1-100 dilution-blot.tif]

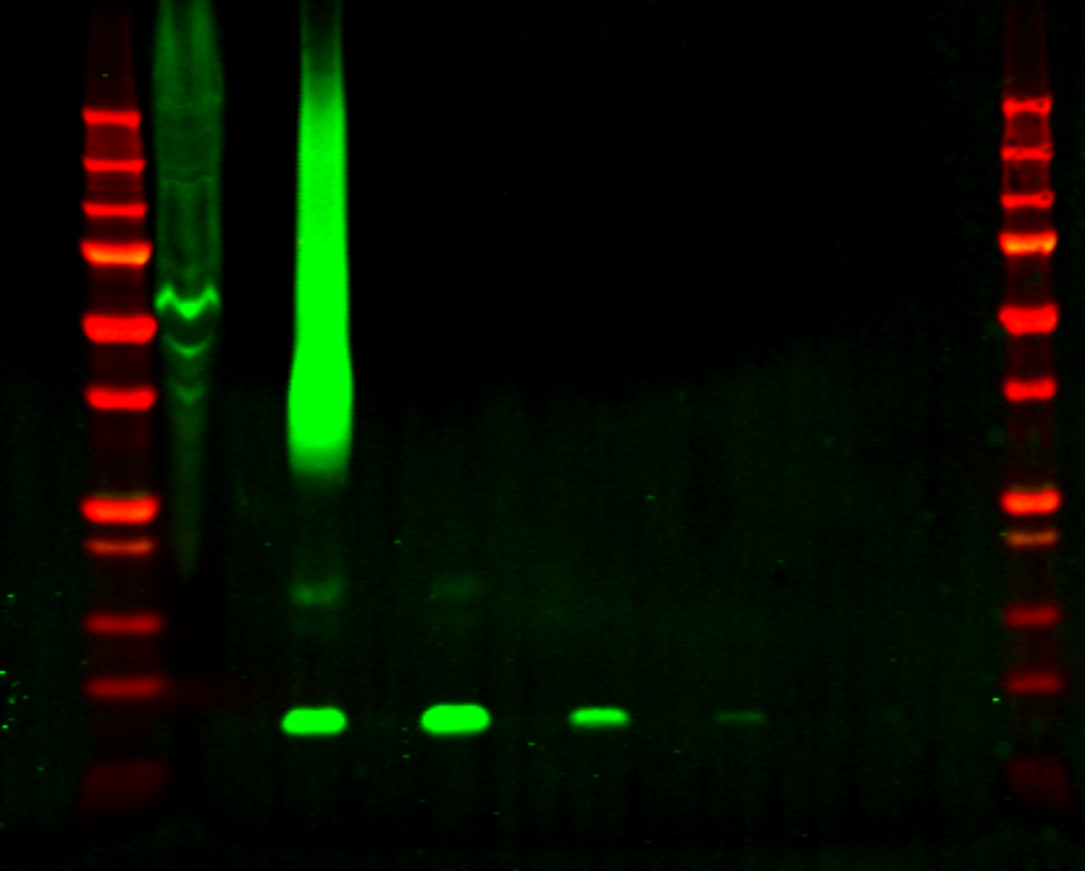

Supplement: S1 File — (ZIP) [file pone.0324280.s001.zip › S1 File/Figure 3 A 1-1000 dilution blot.tif]

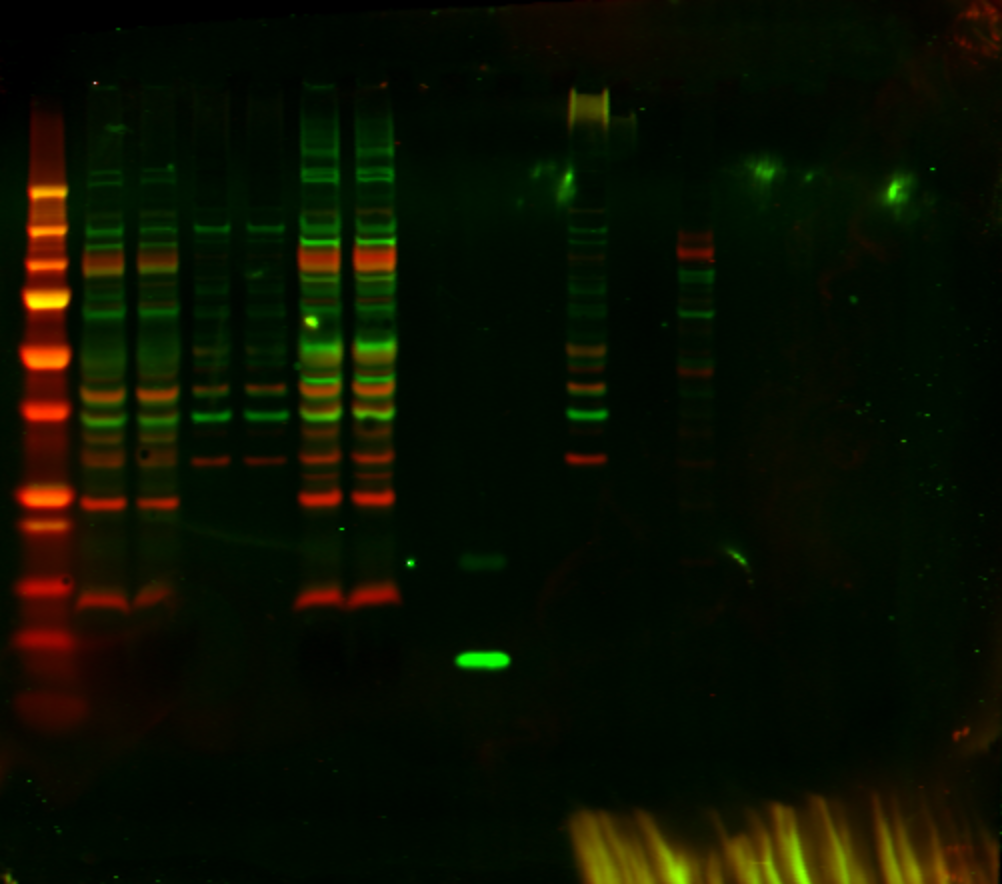

Supplement: S1 File — (ZIP) [file pone.0324280.s001.zip › S1 File/Figure 3B blot.tif]

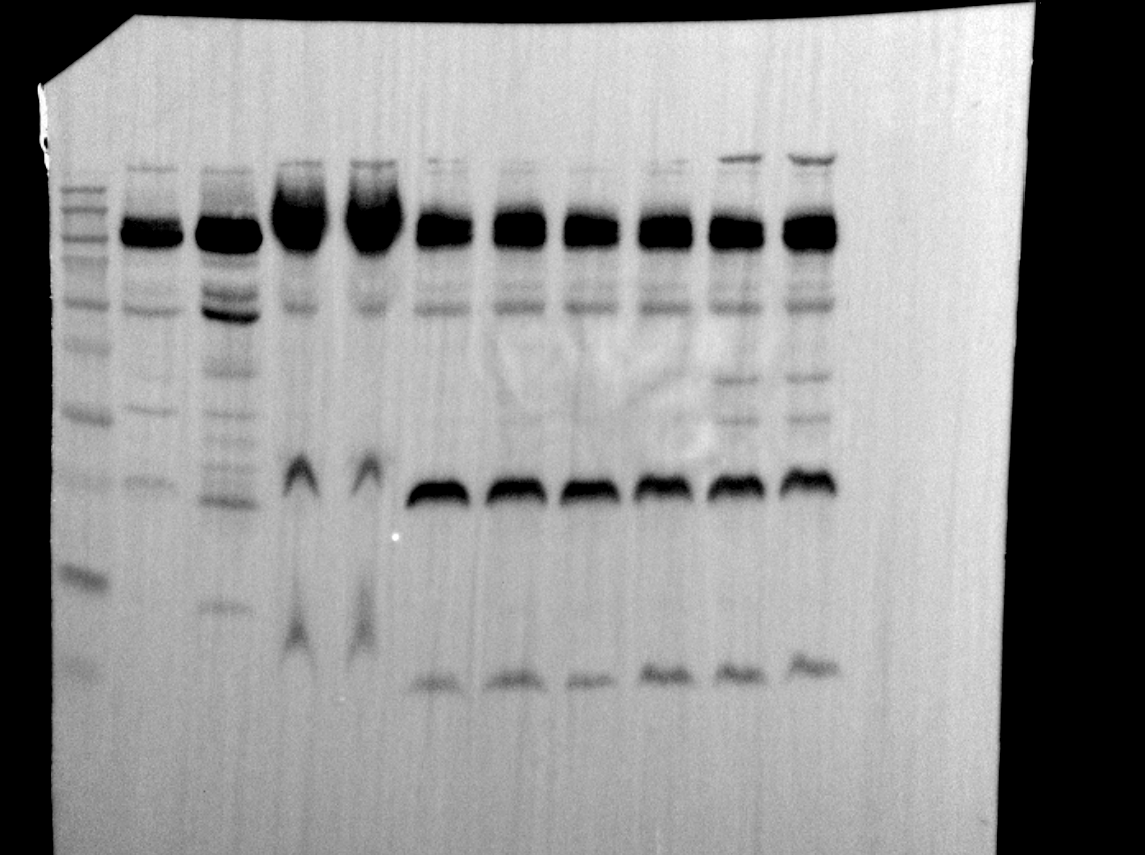

Supplement: S1 File — (ZIP) [file pone.0324280.s001.zip › S1 File/Figure 3C-Anti-APP blot.tif]

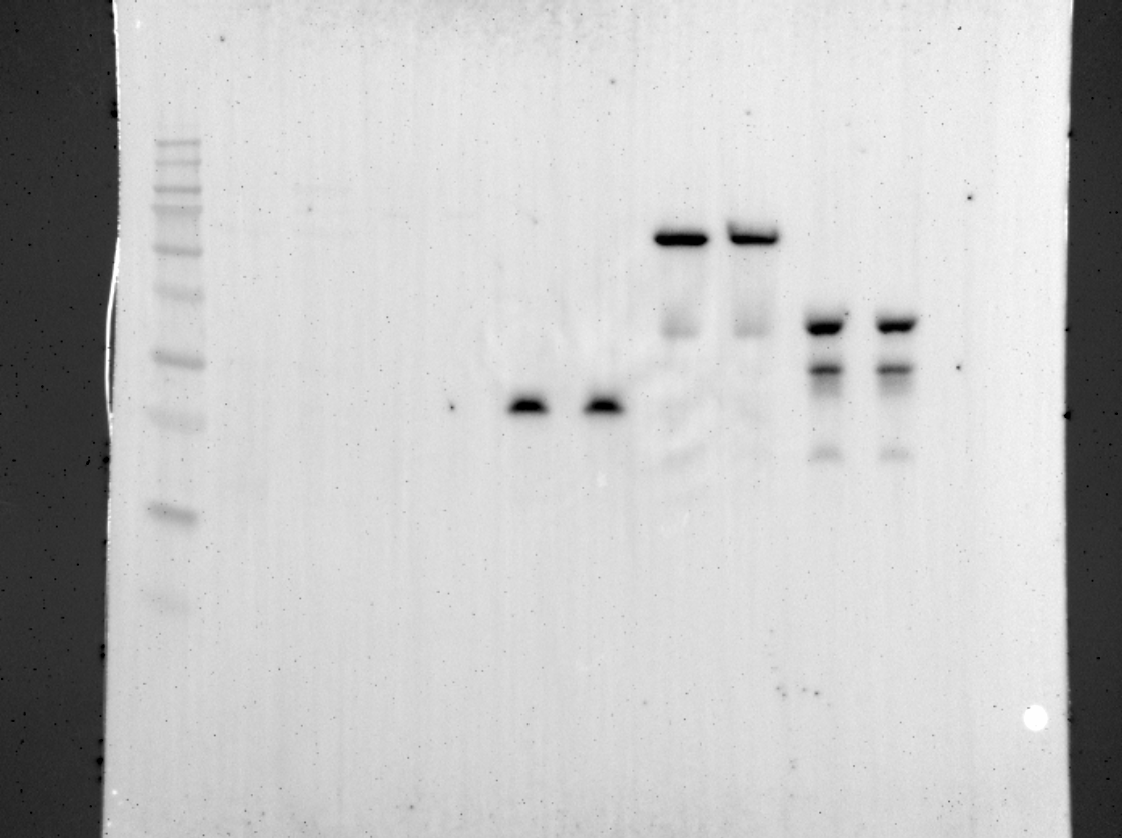

Supplement: S1 File — (ZIP) [file pone.0324280.s001.zip › S1 File/Figure 3C-Anti-GFP blot.tif]

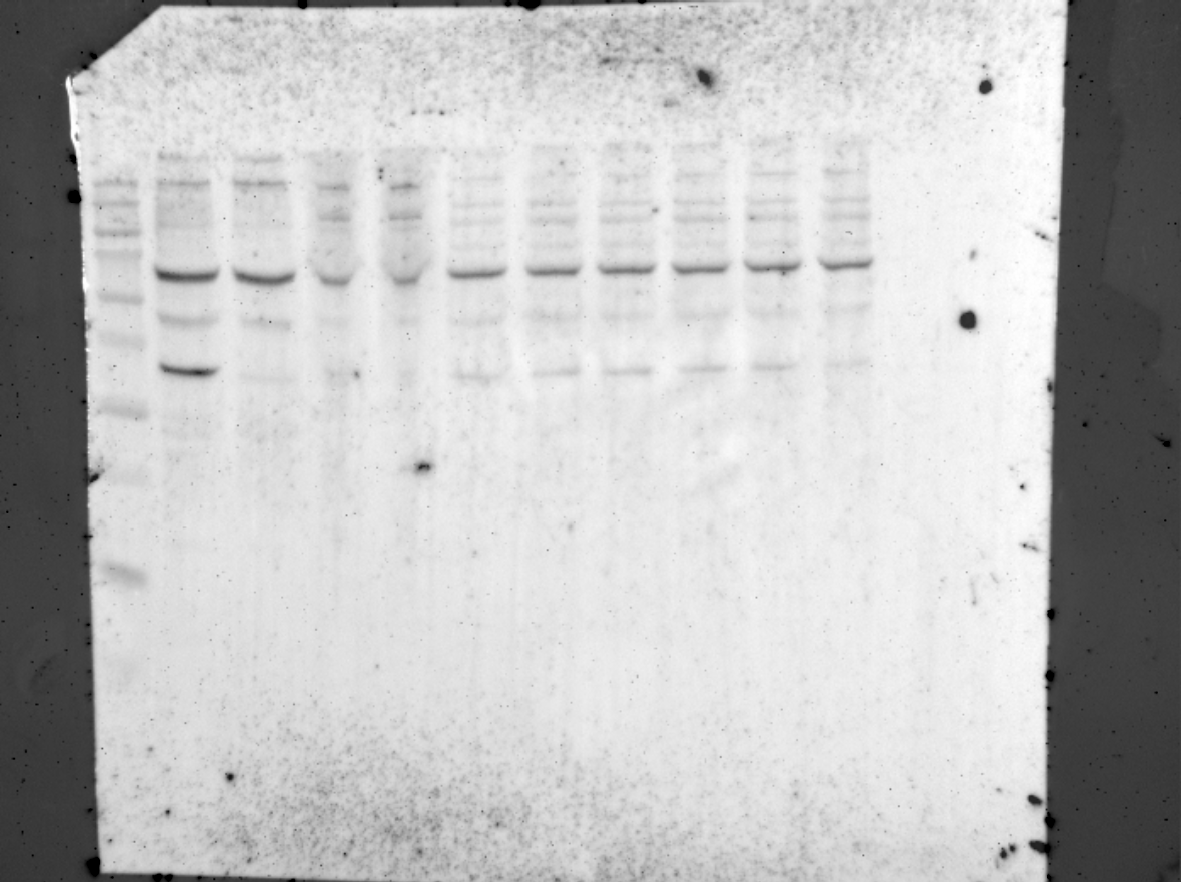

Supplement: S1 File — (ZIP) [file pone.0324280.s001.zip › S1 File/Figure 3C-serum -blot.tif]

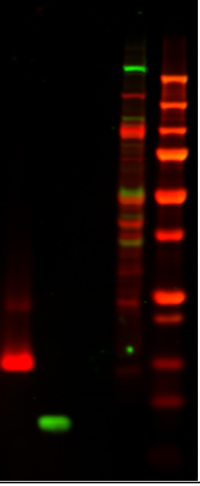

Supplement: S1 File — (ZIP) [file pone.0324280.s001.zip › S1 File/Figure 3D-anti-APP.tif]

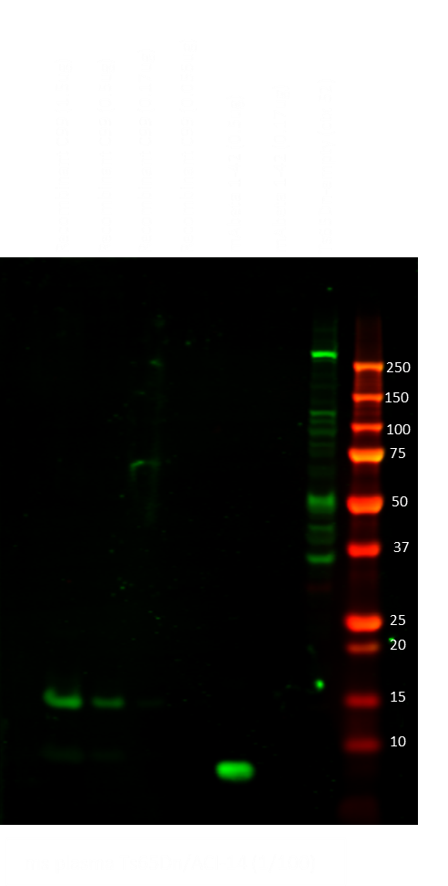

Supplement: S1 File — (ZIP) [file pone.0324280.s001.zip › S1 File/Figure 3D-vaccine blot.tif]

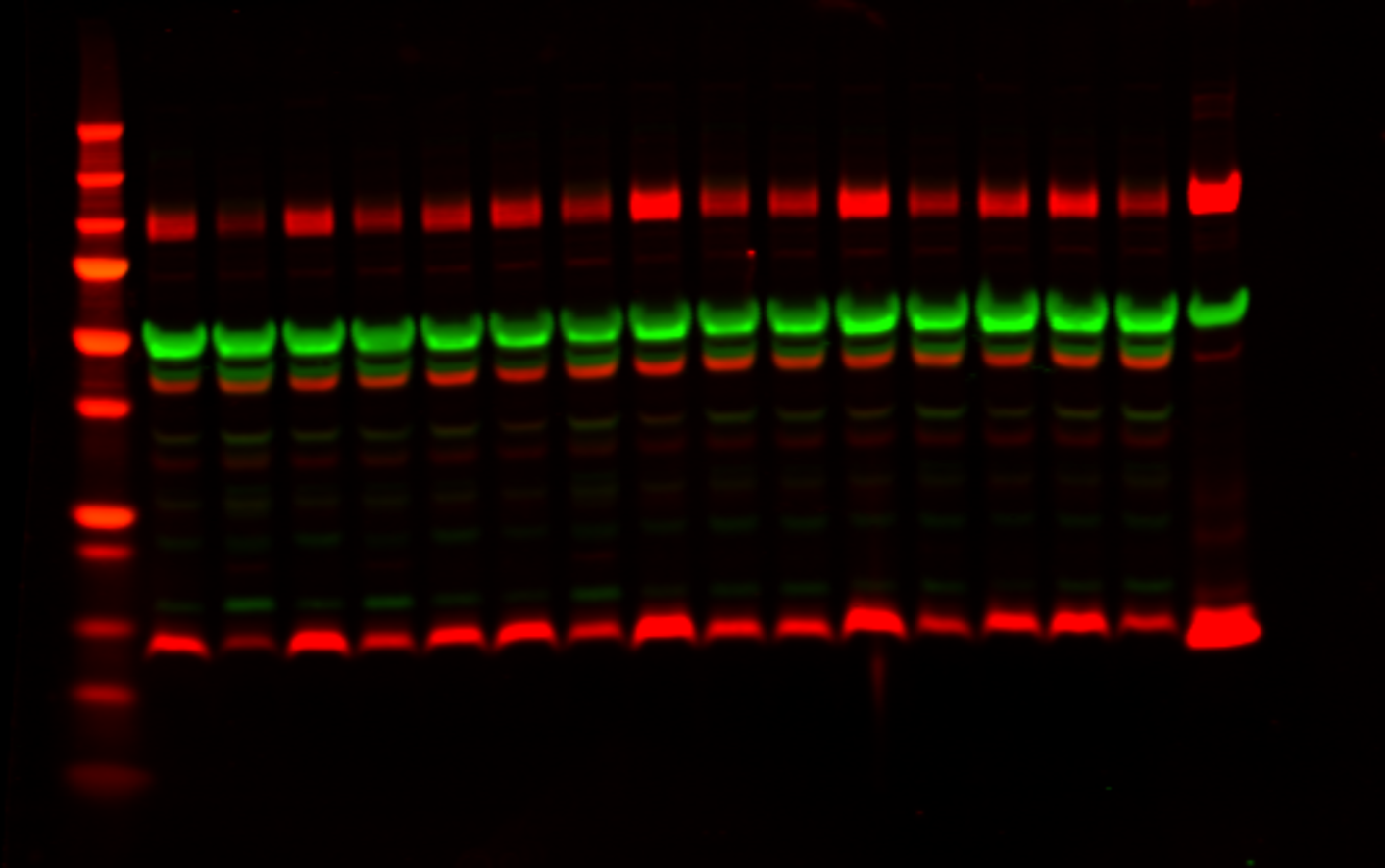

Supplement: S1 File — (ZIP) [file pone.0324280.s001.zip › S1 File/Figure 5 A blot.tif]

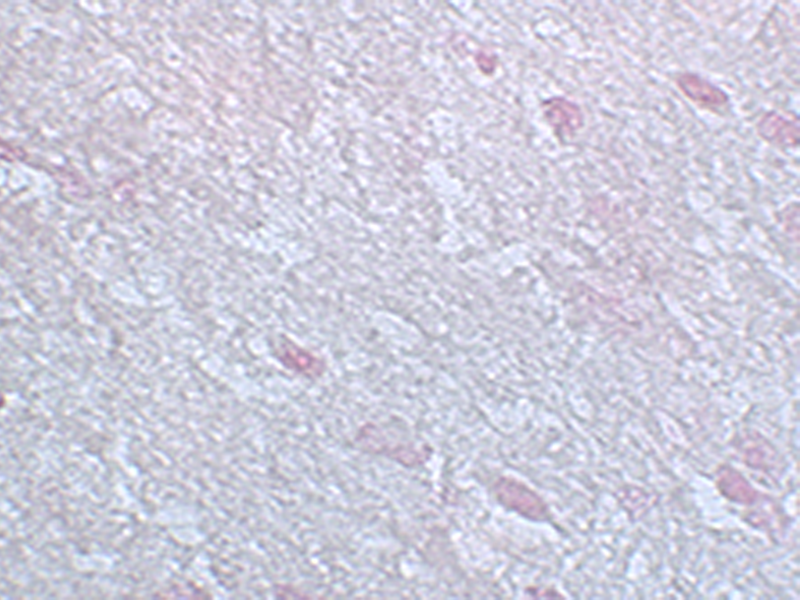

Supplement: S2 File — (ZIP) [file pone.0324280.s002.zip › S2 File/Underlying image original Figure 8E Perls Prussian blue​ - 2N DS-01.tif]

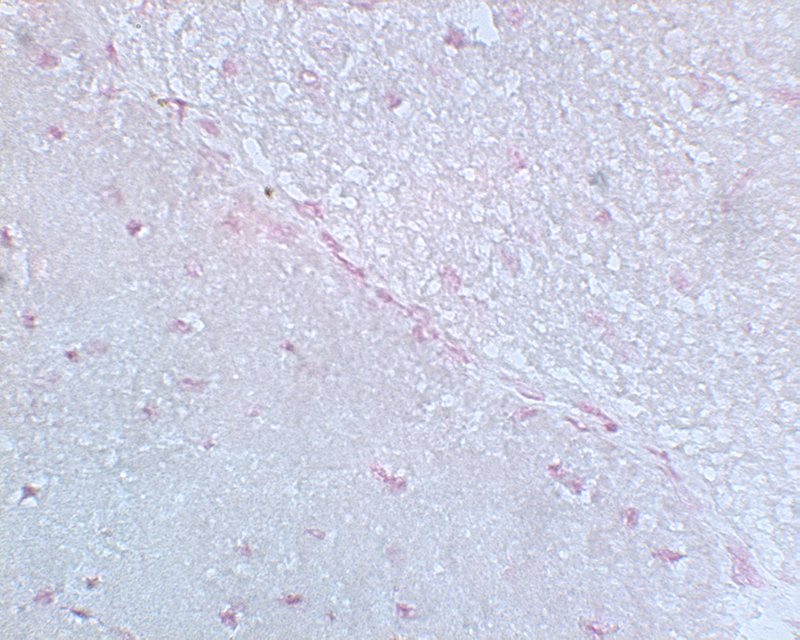

Supplement: S2 File — (ZIP) [file pone.0324280.s002.zip › S2 File/Underlying image original Figure 8E Perls Prussian blue​ - 2N Vehicle.tif]

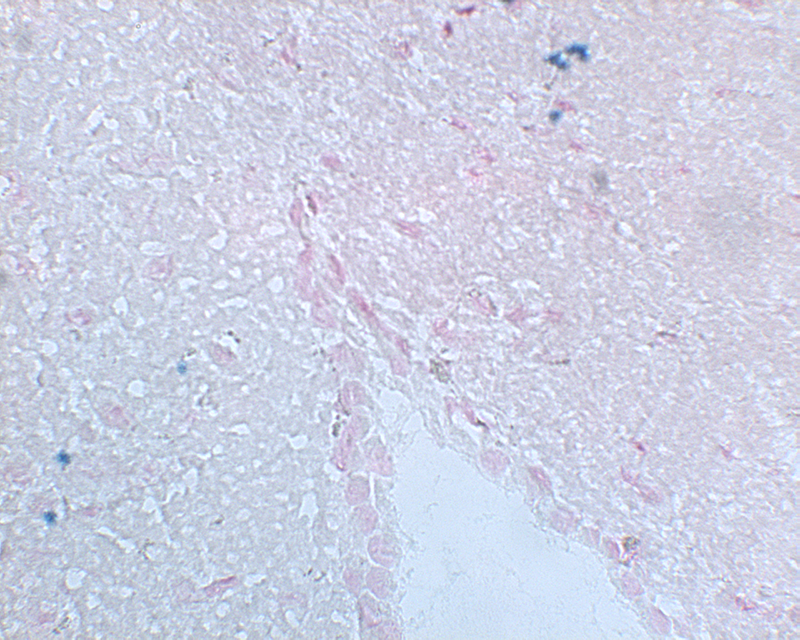

Supplement: S2 File — (ZIP) [file pone.0324280.s002.zip › S2 File/Underlying image original Figure 8E Perls Prussian blue​ - Ts65 DS-01.tif]

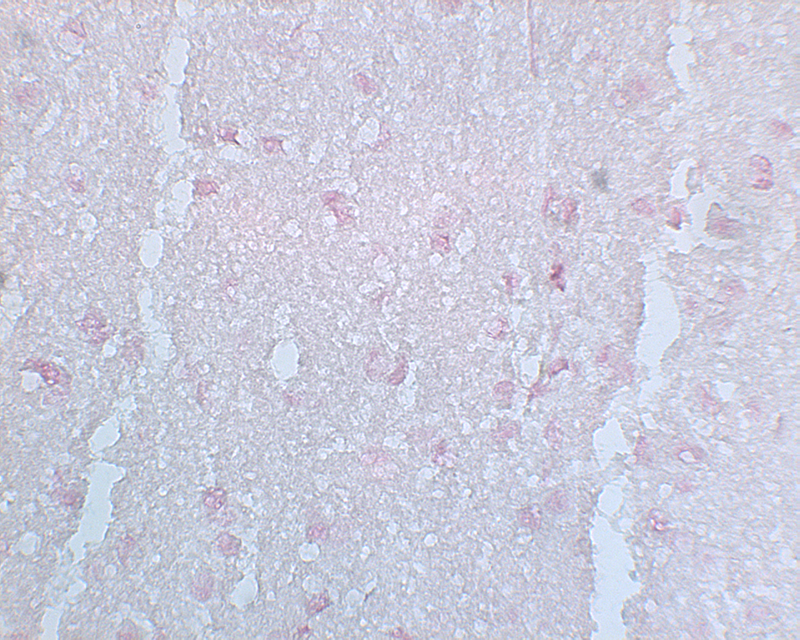

Supplement: S2 File — (ZIP) [file pone.0324280.s002.zip › S2 File/Underlying image original Figure 8E Perls Prussian blue​ - Ts65Dn vehicle.tif]

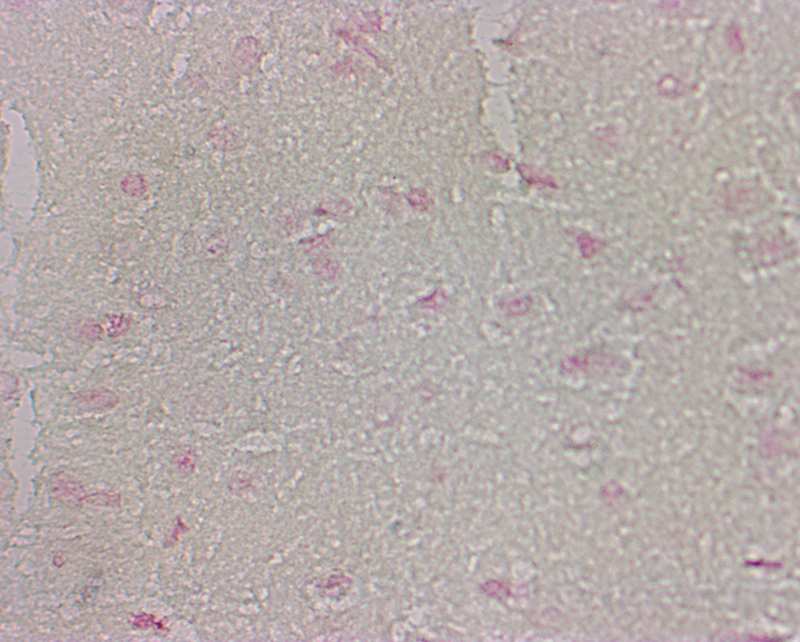

Supplement: S2 File — (ZIP) [file pone.0324280.s002.zip › S2 File/Updated Figure 8E Perls Prussian blue - 2N Vehicle.tif]

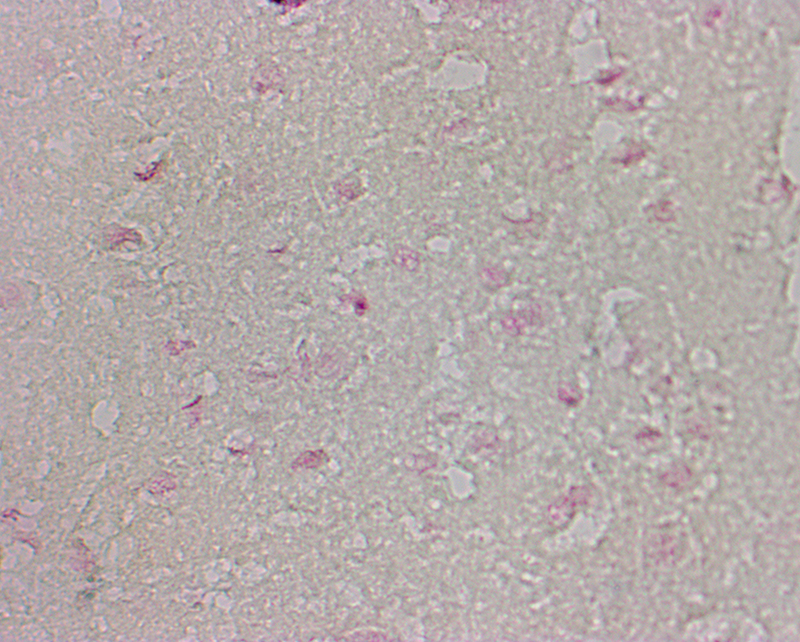

Supplement: S2 File — (ZIP) [file pone.0324280.s002.zip › S2 File/Updated Figure 8E Perls Prussian blue - Ts65Dn DS-01.tif]

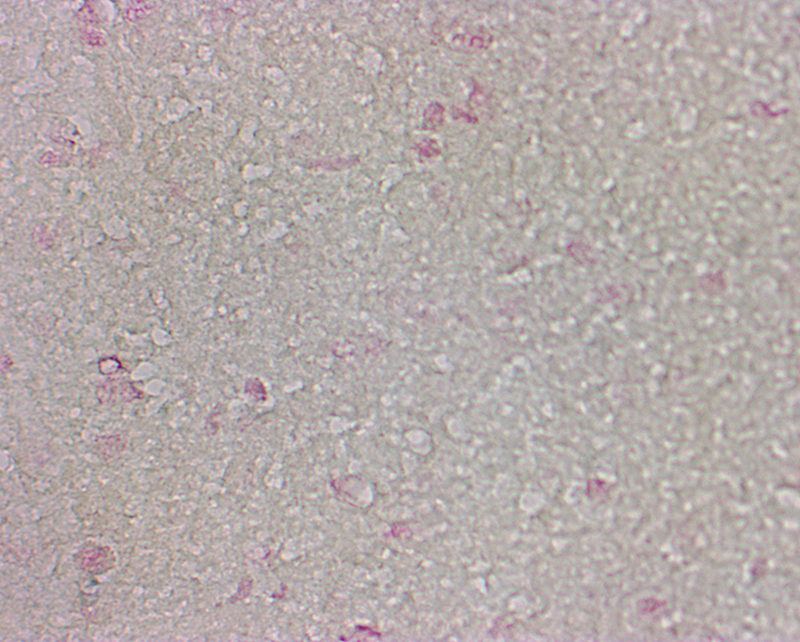

Supplement: S2 File — (ZIP) [file pone.0324280.s002.zip › S2 File/Updated Figure 8E Perls Prussian blue - Ts65Dn Vehicle.tif]

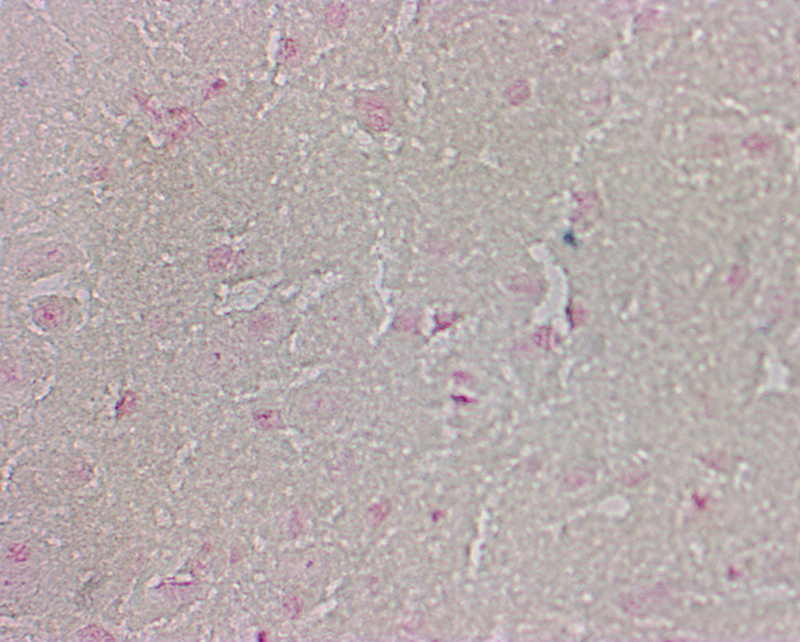

Supplement: S2 File — (ZIP) [file pone.0324280.s002.zip › S2 File/Updated Figure 8E Perls Prussian blue- 2N DS-01.tif]

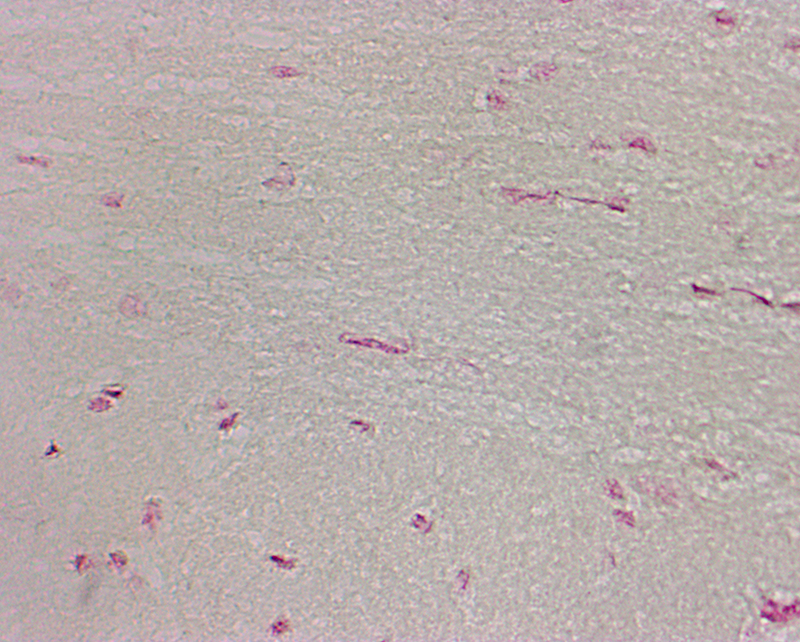

Supplement: S2 File — (ZIP) [file pone.0324280.s002.zip › S2 File/W13-1699_A3_2N _vehicle_slide03.tif]

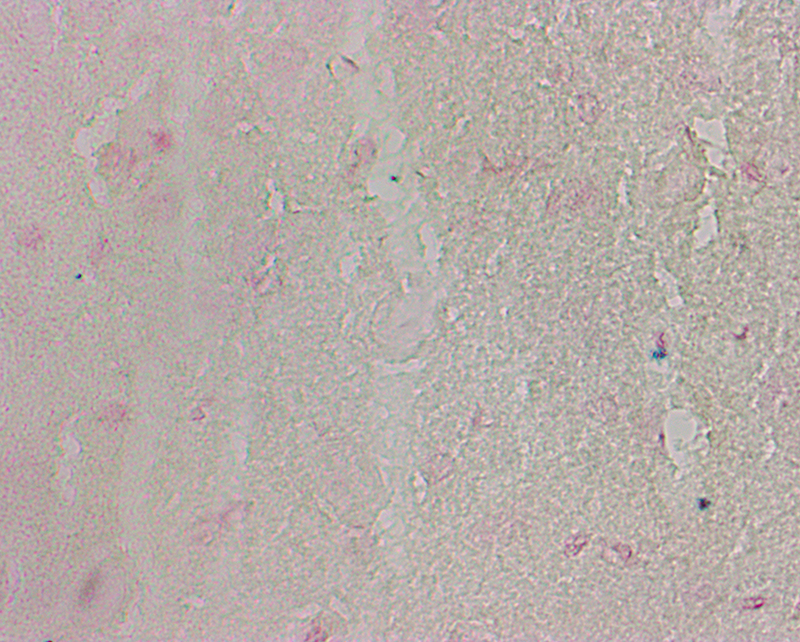

Supplement: S2 File — (ZIP) [file pone.0324280.s002.zip › S2 File/W13-1699_A7_2N_vehicle_slide02.tif]

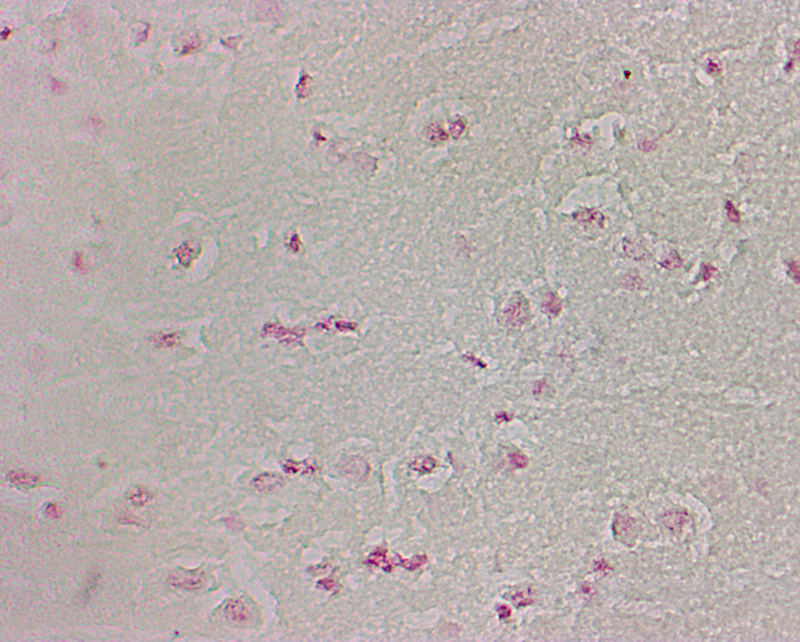

Supplement: S2 File — (ZIP) [file pone.0324280.s002.zip › S2 File/W13-1699_B07_2N_DS01_slide_02.tif]

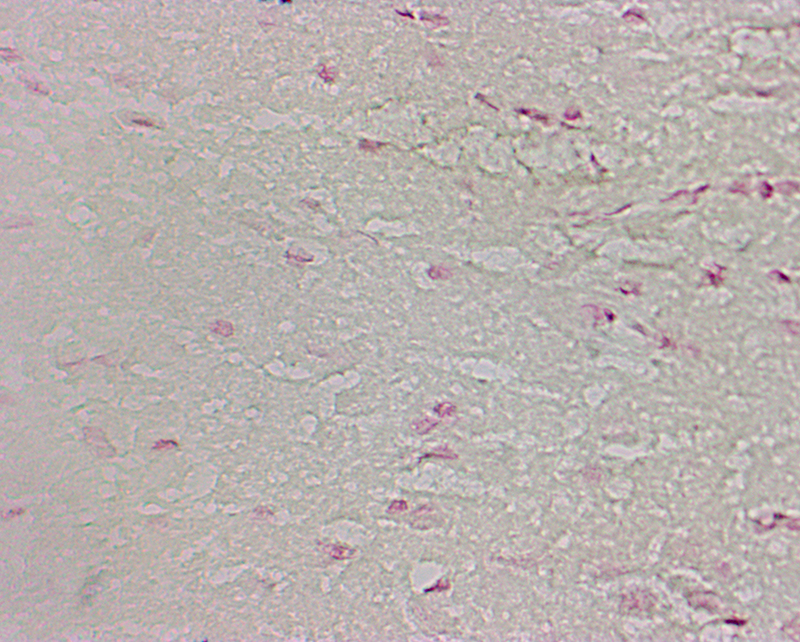

Supplement: S2 File — (ZIP) [file pone.0324280.s002.zip › S2 File/W13-1699_B08_2N_DS01_slide_02.tif]

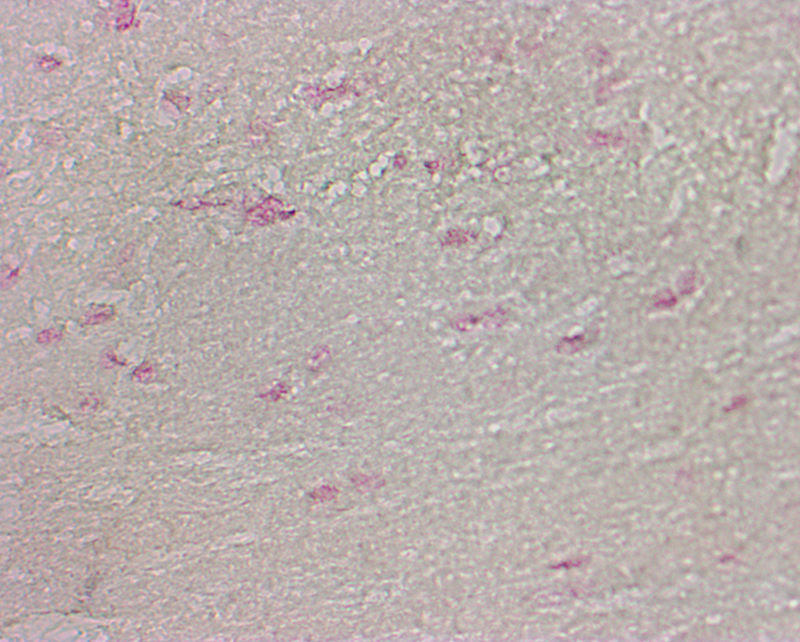

Supplement: S2 File — (ZIP) [file pone.0324280.s002.zip › S2 File/W13-1699_C1_Ts65Dn-_vehicle_slide_03.tif]

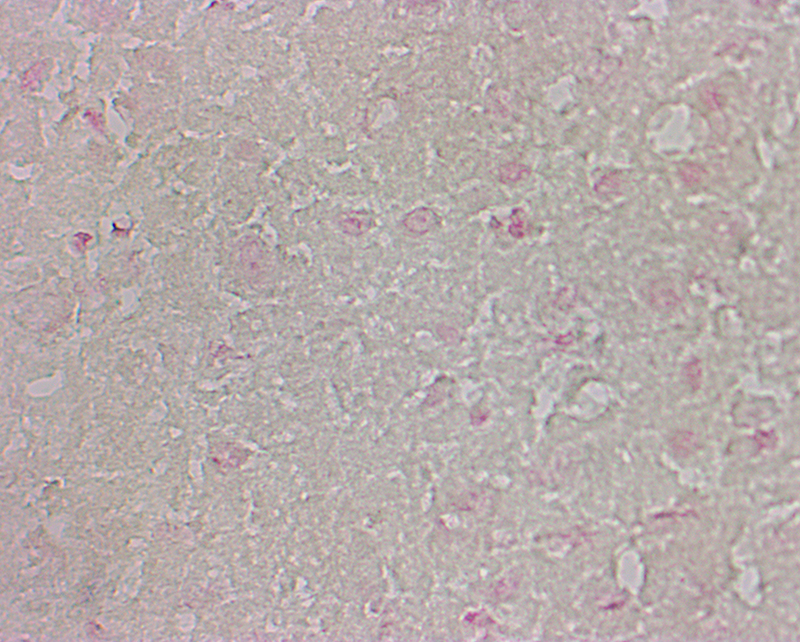

Supplement: S2 File — (ZIP) [file pone.0324280.s002.zip › S2 File/W13-1699_C3_Ts65Dn-_vehicle_slide_03.tif]

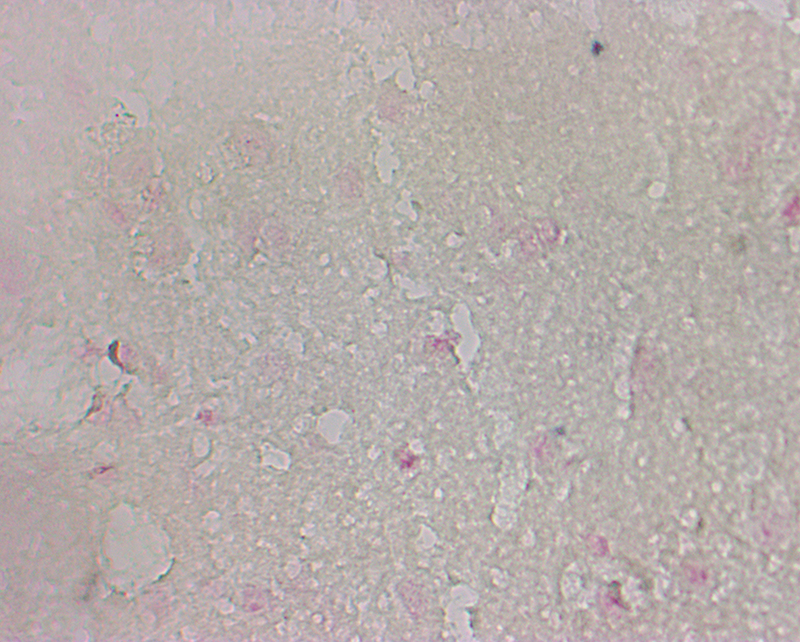

Supplement: S2 File — (ZIP) [file pone.0324280.s002.zip › S2 File/W13-1699_D4_Ts65Dn_DS01_slide_02.tif]

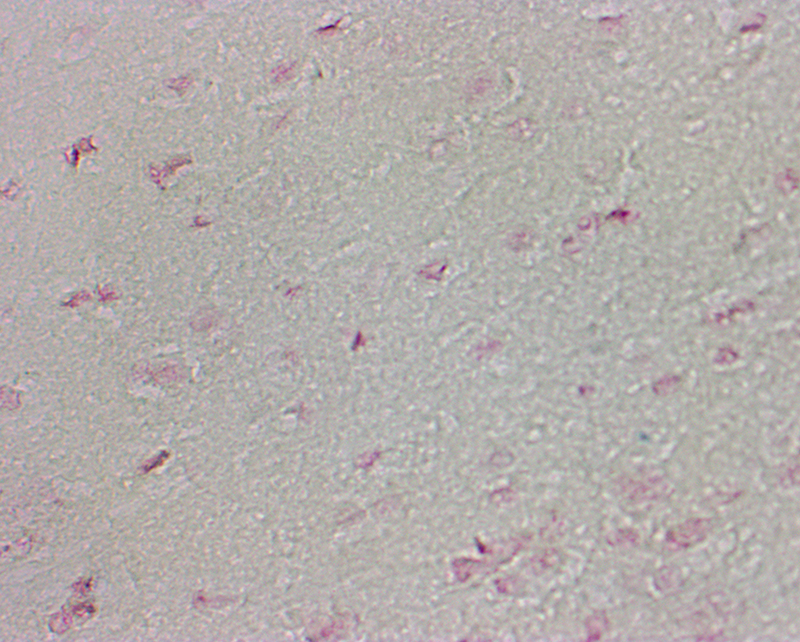

Supplement: S2 File — (ZIP) [file pone.0324280.s002.zip › S2 File/W13-1699_D6_Ts65Dn_DS01_slide_02.tif]
